# Supplementary material for: Fungal Assemblages in Northern Elms—Impacts of Host Identity and Health, Growth Environment, and Presence of Dutch Elm Disease
Source: Microb Ecol. 2025 Jul 24;88:82. doi: 10.1007/s00248-025-02585-2 (PMC12289760; doi:10.1007/s00248-025-02585-2)
Supplement: Supplementary file 1 [file 248_2025_2585_MOESM1_ESM.pdf]

# Fungal ~~a~~Assemblages in ~~n~~Northern ~~e~~Elms~~—~~—~~i~~Impacts of ~~h~~Host ~~i~~Identity and ~~h~~Health, ~~g~~Growth ~~e~~Environment, and ~~p~~Presence of Dutch ~~e~~Elm ~~d~~Disease

Liina Jürisoo[1,2*], Ahto Agan[1], Leho Tedersoo[3,4], Johanna Witzell[2], Andrey Selikhovkin[5], Rein Drenkhan[1]

[1] Institute of Forestry and Engineering, Estonian University of Life Sciences, Tartu, Estonia~~;~~
[2] Department of Forestry and Wood Technology, Linnaeus University,~~;~~ Växjö, Sweden
[3] Mycology and Microbiology Center, University of Tartu, Tartu, Estonia~~;~~
[4] Institute of Ecology and Earth Sciences, University of Tartu, Tartu, Estonia~~;~~
[5] Department of Forest Protection, Wood Science and Game Management, Saint Petersburg State Forest Technical University, Saint Petersburg, Russia~~.~~

*Corresponding author email: liina.jyrisoo@emu.ee

## Abstract

Dutch elm disease (DED), caused by the pathogenic ascomycete *Ophiostoma novo-ulmi*, has devastated natural elm (*Ulmus* spp.) populations in Europe and North America. Elm species vary in their susceptibility to this vascular disease, which may partly reflect differences in their associated mycobiomes. To investigate the diversity and composition of fungal endophyte communities in relation to host genotype, health status, and environment, we analy~~s~~zed twig-associated fungi in symptomatic and asymptomatic individuals of highly susceptible *U. glabra*, less susceptible *U. laevis*, and hybrid elms growing in Estonia and Russia. Fungal communities were analy~~s~~zed using PacBio long-read amplicon sequencing of the ITS1-5.8S-ITS2 gene region.

Tree species exhibited distinct fungal community profiles. *Ophiostoma novo-ulmi* was detected exclusively in symptomatic trees and was dominant in *U. glabra*~~,~~; it was absent in symptomatic hybrid elms. In contrast, the canker-associated pathogen *Sphaeropsis ulmicola* occurred in both symptomatic and asymptomatic trees, was dominant in symptomatic *U. laevis*, and common in symptomatic and healthy *U. glabra*, though less prevalent in symptomatic hybrid elms. Remarkably, *S. ulmicola* was associated with the highest level of damage ~~of~~in *U. laevis*~~,~~ while being present also in hybrid elms. While *O. novo-ulmi*'s presence did not affect overall fungal richness, *S. ulmicola* was linked to higher fungal diversity. Additionally, fungal species richness was significantly greater in urban compared to rural environments. This was the first comparable ~~analyzse~~analysis of fungal diversity and composition on three different *Ulmus* species shoots.

**Key~~-~~words**: *Ophiostoma novo-ulmi*;~~,~~ ~~m~~Metabarcoding;~~,~~ ~~m~~Mycobiome;~~,~~ *Sphaeropsis ulmicola*;~~,~~ *Ulmus* spp.;~~,~~ ~~i~~Invasive pathogen

## Introduction

Fuelled by the increasing interest in microbe-based plant protection and the advancements of molecular tools, numerous studies have recently examined foliar endophytic fungal communities across tree species. These include research on both conifers [1-4] and deciduous trees, such as ash (*Fraxinus* spp.) [5-7] and elms (*Ulmus* spp.) [8-10]. Endophytes~~—~~—microorganisms living harmlessly within plant tissues~~—~~—play vital roles in tree health, including in elms. They help trees tolerate abiotic stresses like drought, salinity, and temperature extremes by producing phytohormones, antioxidants, and other beneficial compounds [11]. Emerging evidence suggests they also contribute to disease resistance, a potentially critical function in long-lived forest trees [12-14]. However, the diversity and dynamics of tree-associated fungal communities remain poorly understood.

Elms (*Ulmus* spp.) are deciduous trees commonly found in riparian forests of temperate regions across the Northern Hemisphere [15]. Throughout their range, native elm trees play an important ecological role, providing ecosystem services and supporting a variety of organisms, including lichens and fungi [16-19]. Elms are also valued as ornamental trees and are widely planted in urban and suburban environments [20, 21]. The native elm species affected by Dutch elm disease (DED) in Estonia and Russia are *U. glabra* and *U. laevis.* These regions are near the northern border of their natural range [15, 22]. Unfortunately, pandemics of DED, caused by fungal pathogens in the genus *Ophiostoma*, have led to substantial losses of elm trees across Europe [23] and North America [24]. As a result, hybrid elm cultivars have been planted in urban areas since the early 2000s [25] to help restore elm populations and improve disease resistance.

Given the complex interactions between elm hosts and their associated pathogens, resistance to DED is not uniform across *Ulmus* species. For example, ~~while Although~~although *U. laevis* has not been a primary target of breeding programs, the widespread decline of elms has nonetheless

prompted interest in identifying or preserving naturally DED-tolerant genotypes within elms, which may help to preserve the ecological roles [26, 27].

The presence of DED in Estonia and Russia ~~DED~~ has been recorded since the 1930s [25, 28, 29]. The pathogen is transmitted by elm bark beetles from the genera *Scolytus* [30], *Hylurgopinus* [31], *Xyleborus*, and *Xyleborinus* [32] or via root grafts [33]. *Ophiostoma* fungi proliferate in the tree's vascular system [34], causing wilting and often death. As a result of DED, native elm populations have declined rapidly, and in countries such as Sweden, native elms are now listed as critically endangered [35].

Intriguingly, our recent observations indicate that DED is not the only cause of ~~-~~dieback in elms in Estonia [36]. We found S*phaeropsis ulmicola* to be commonly present in the shoots of elm trees showing wilting symptoms similar to DED [36]. *Sphaeropsis ulmicola* (syn. *Botryodiplodia ulmicola*) has been reported as a shoot canker in the U~~nited~~ S~~tates~~A [37] and Poland [38]. The infection may contribute to progressive dieback in elm saplings over time, particularly because unhealed cankers serve as entry points for secondary pathogens~~.~~ [38, 39].

Recent research highlights the importance of the holobiont~~—~~—the host and its associated microbiota functioning as a single unit~~—~~—in understanding disease resistance [40-46]. The microbial communities are influenced by tree genotype, health status, and environmental conditions such as water availability [47-49]. Accumulating evidence indicates that fungal endophytes (mycobiota) can modulate host defen~~c~~ses or inhibit pathogens directly through competition or antifungal compound production [47, 50-52]. Traditional breeding often overlooks the role of plant-associated endophytes, despite their potential influence on host traits. Incorporating the holobiont concept requires new genotyping and phenotyping methods that include microbial communities [53]. High-throughput sequencing (HTS) and other ~~"~~“omics~~"~~” technologies can profile the microbiome associated with plants, providing insights into the functional roles of endophytes [54].

Previous studies have revealed complex relationships between endophyte communities and DED resistance in elms. For instance, Martín et al. [9] reported low diversity and frequency of culturable, xylem-residing endophytic fungi in elms showing low susceptibility to DED,

suggesting that some endophytes may be suppressed by the same resistance mechanisms that target the pathogen. More recently, Macaya-Sanz et al. [55] employed culture-independent methods and identified three yeast families (Buckleyzymaceae, Trichomeriaceae, and Bulleraceae) associated with DED resistance in *U. minor*. Furthermore, Marčiulynas et al. [8] observed a negative relationship between DED presence and fungal diversity in leaves and roots of young (*ca.* 26–38 years old) *U. glabra* trees.

Recent health assessments of native elms in ~~the~~ northeastern Europe have shown that *U. laevis*, which is more common in the eastern parts of the region [15], is generally healthier than *U. glabra* [25, 28]. Similarly, various hybrid elms (e.g., *Ulmus davidiana* var. *japonica* × *U. pumila* 'New Horizon') that are expected to be healthier have been imported and planted ~~to~~in the Nordic areas. However, the hybrid elms also face problems, and issues such as dead shoots or partly damaged canopy have been observed, warranting further analysis. Isolation-based studies have also revealed a rich diversity of endophytes in the twigs of these elm species [25, 28]. However, since culturing only ~~capture~~captures a fraction of fungal diversity, it remains unclear to what extent differences in endophytic community composition and richness explain the observed phenotypic variation.

In this study, we investigated the poorly characterized twig-associated endophytic communities in northern populations of *U. glabra*, *U. laevis*, and hybrid elms. Our objectives were fourfold: (1) to determine whether fungal abundance, diversity, and putative functional traits differ among symptomatic and asymptomatic trees of *U. glabra*, *U. laevis*, and *Ulmus* hybrids; (2) to explore differences in community composition between healthy and symptomatic individuals within species or hybrid groups; (3) to assess the influence of environmental context by comparing endophyte communities in urban versus rural sites; and (4) to evaluate whether differences in mycobiota composition are associated with the presence of *Ophiostoma novo-ulmi* and *Sphaeropsis ulmicola*, a commonly observed co-occurring fungus. To address these questions, we collected twig samples from elm trees in Estonia and northwestern Russia and used high-throughput sequencing to characterize fungal diversity.

## Material and ~~m~~Methods

*Study Sites and Sampling*

A total of 183 mature *U. glabra*, *U. laevis*, and *Ulmus* hybrids (elm hybrids) trees were identified by species and variety using the identification key of Hillier Nurseries [40], across 11 sites located in rural and urban areas in Estonia and urban environments in the Leningrad Region, Russia (Fig. 1, Table 1). The health status of the trees was assessed according to Jürisoo et al. [28]. In brief, five general crown vitality classes were determined by visual assessment: (1) healthy; (2) minor; (3) medium; (4) major crown damage; (5) dead tree.

Using telescopic secateurs, one current year shoot (~25 cm long, collected at a height of 1–4 m) was harvested from each tree in 2015 and 2016 (see Supplementary Table 5). Asymptomatic branches were collected from healthy trees or from trees with minor damage (vitality class 2), while symptomatic branches—displaying typical DED symptoms such as brown rings or dots under the bark—were collected from trees in vitality classes 3 and 4. The secateurs were sterilized after each cut. Each sample was individually packed into a labeled sterile plastic bag, transported to the laboratory, and stored at –20 °C.

**Table 1** Number of surveyed and sampled elm trees in northeastern Europe (Estonia and Russia)

| Country | Total | Species | | | | | |
|---|---|---|---|---|---|---|---|
| | | *Ulmus glabra* | | *Ulmus laevis* | | *Ulmus* hybrids[1] | |
| | | Symptomatic | Healthy | Symptomatic | Healthy | Symptomatic | Healthy |
| Estonia | 114 | 50 | 24 | 11 | 20 | 5 | 4 |
| Russia | 69 | 19 | 6 | 14 | 4 | 19 | 7 |
| No. of trees | 183 | 69 | 30 | 25 | 24 | 24 | 11 |

[1] *Ulmus davidiana* var. *japonica* × *U. pumila* 'New Horizon' (nine surveyed trees in Estonia), unknown hybrids in Russia. The proportion of positive detection of DED was determined by sequencing

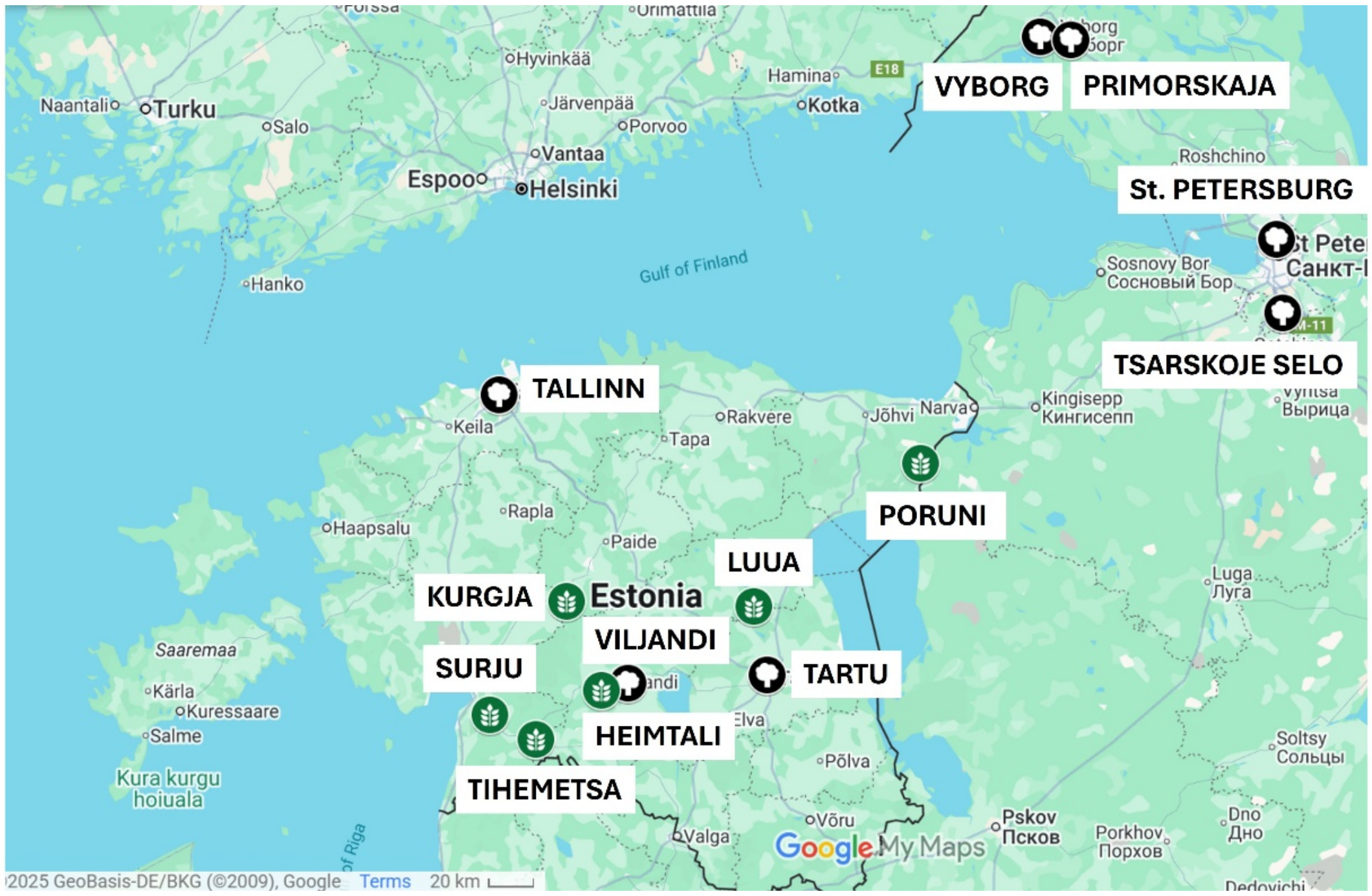


urban environments countryside

**Fig. 1**~~.~~ Sampling sites in Estonia and Russia~~.~~

*Molecular ~~a~~Analysis*

The samples for DNA extraction were prepared by peeling bark from the shoots using a sterile scalpel. Small pieces from xylem (~2 g) were placed into sterile Eppendorf tubes and stored in ~~-~~–20 ~~□~~°C until DNA extraction (*n* = 183). DNA was extracted using the GeneJET Genomic DNA purification kit (Thermo Fischer Scientific, Vilnius, Lithuania) following Jürisoo et al. [28]. Fungal DNA was amplified using primers ITS4ngs [56] and ITS1catta [57]. The ITS4ngs primer included a 10–12 base multiplex identifier (MID) index that differed from any of the 107 other indices by at least four bases.

Conventional PCR was performed in two replicates for each sample, in a 25 µl reaction volume containing 0.5 µ~~µ~~l each of forward and reverse primers, ~~and~~ 5 µ~~µ~~l of HOT FIREPol Blend Master Mix Ready to Load (Solis BioDyne, Tartu, Estonia), 1 µl of sample DNA, and 18 µl of DNA-free water. Amplification conditions were~~:~~ 15 min at 95 °C, followed by 25 cycles of 30 s at 95 °C, 30 s at 55 °C, 1 min at 72 °C, and a final step at 72 °C for 10 min. PCR products were visualized on 1% agarose gels. Samples without visible bands were reamplified using up

to 35 cycles. Products were purified with the GeneJET~~et~~ DNA purification kit (Thermo Fischer) following the manufacturer’s instructions.

Amplicons were pooled into two sequencing libraries (one per country) in equimolar ratios. Library preparation followed protocols for the PacBio third-generation sequencing platform (Pacific Biosciences, Inc. Menlo Park, CA, USA). Libraries were loaded to SMRT cells using the diffusion method and sequenced for 10 h~~ours~~ using P6-C4 chemistry, following Tedersoo et al. [58]. Sequencing was conducted on the PacBio RSII platform at the University of Oslo Sequencing Centre.

### *Bioinformatics ~~a~~Analysis*

Bioinformatics was performed using tools integrated in Pipe~~c~~Craft 1.0 [59]. Reads shorter than 100 bp were removed using mothur [60]. Longer sequences were demultiplexed, allowing for one-base mismatch in the index and two in the primer. De novo chimera filtering was done with UCHIME [61].The full-length Internal Transcribed Spacer (ITS) region was extracted using ITSx. Sequences were clustered into Operational Taxonomic Units (OTUs) at 99% similarity using CD-HIT [62]. The remaining OTUs were taxonomically assigned based on representative sequences against the UNITE v. 9.0 database [63]~~.~~, classified as fungi if their best BLAST hit was a fungal taxon with an *e*-value < *e*−50. Representative sequences with >99% similarity to reference sequences were assigned to Species Hypotheses (SHs) according to UNITE. Higher-level fungal taxonomy followed the *e*-value and similarity criteria of Tedersoo et al. [56].

### *Statistical ~~a~~Analysis*

OTU richness was calculated for each sample using PAST3 [64]. Rarefaction analysis was also performed in PAST3 to evaluate whether sampling effort was sufficient to capture the majority of OTUs. Linear mixed models were used to test the effects of tree species and health status on overall taxonomic richness, diversity, and *O. novo-ulmi* abundance, using the lme4 package in R (version 4.2.2) [65]. Habitat was included as a random intercept, and the square root of the total number of sequences per sample was used as a covariate. Differences in the abundance of

dominant fungal taxa across multiple factors were assessed via ANOVA followed by Tukey's HSD post hoc test.

Functional group assignment of species was based on the FungalTraits database [66]. The final results were manually cross-checked on a species level as genus-based trait assignment can lead to false functional groupings of some species, e.g., when some of the species in one genus are endophytes while others are clearly plant pathogens. Differences in fungal community composition between countries, environments (urban vs. rural), elm species, and health statuses (symptomatic vs. healthy) were tested using PERMANOVA+ [67], based on Bray-Curtis dissimilarity of square root-transformed OTU abundances. Principal ~~C~~coordinates ~~A~~analysis (PCoA) was used to visualize fungal community structure in Primer v6 [68]. Species co-occurrence analysis was conducted using the co-occur function in R [69] to detect species that were positively or negatively associated across the dataset. Co-occur function uses ~~presence absence~~presence-absence data to estimate pairwise species associations as it calculates both observed and estimated co-occurrence and uses a hypergeometric distribution to test ~~are~~ whether ~~the~~ observed co-occurrence patterns are higher than expected~~,~~ (a positive association), lower than expected~~,~~ (a negative association), or not significantly different~~,;~~ (association is random). These analyses were carried out separately for each country.

## Results

### *Overall ~~c~~Characteristics of ~~f~~Fungal ~~a~~Assemblages in ~~e~~Elm ~~t~~Twigs*

After removing singletons and low-quality reads, the dataset comprised ~~14 807~~14,807 high-quality ITS1-5.8S-ITS2 sequences from 183 samples, encompassing 305 OTUs. Of all the sequences in the dataset, 87.5% were assigned to Ascomycota, 6.3% to Basidiomycota, and 6.2% remained unidentified (Supplementary Material, Table S1). The rarefaction curve (Fig. S1) did not reach a plateau, indicating that additional sampling would likely increase taxonomic coverage.

Dothideomycetes was the dominant class in all elm species, with a sequence abundance varying from nearly 40% in the hybrids up to about 55% in *U. glabra*. Sordariomycetes were the second most common class (sequence abundance 25.7%), followed by fungi in an unknown class

(sequence abundance 6.9%). ~~Twenty~~The twenty most abundant OTUs (Fig. 2; Supplementary Material, Table S2) covered 83.0%, 79.1%, and 84.8 % of all the sequence reads from healthy and symptomatic *U. glabra*, *U. laevis*, and elm hybrids, respectively.

The most abundant functional group of fungi, regardless of the tree taxa or health status, was plant pathogens (42.7 % of all identified taxa), followed by wood saprotrophs and fungi with unknown functional group (15.9 % and 13.5 %, respectively) (Fig. S1). There were no significant differences in relative abundance of different functional groups between the three *Ulmus* taxa (~~*P*~~$p > 0.05$; supplementary Fig. S1), but the percentage of plant pathogens was significantly higher ($F_{1.177} = 4.17$; ~~*P*~~$p = 0.042$) in symptomatic trees (33.8 %) than in healthy trees (23.4 %). The other functional groups had no significant differences between healthy and symptomatic trees (~~*P*~~$p > 0.05$; Fig. S3).

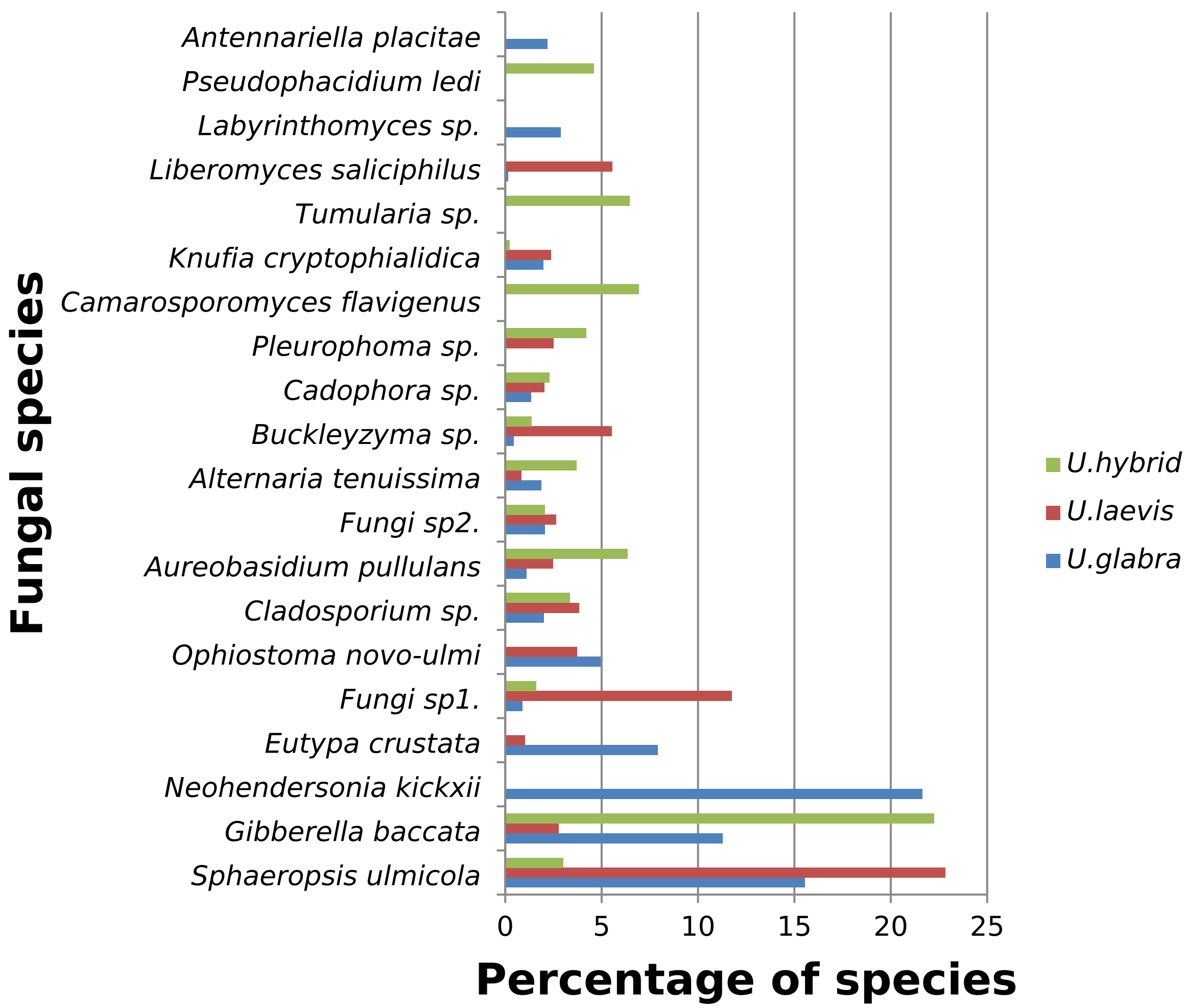


**Fig. 2.** Twenty most abundant taxa in the dataset and their relative abundance on *U. glabra* (*n* = 99), *U. laevis* (*n* = 49) and elm hybrids (*n* = 35)

Across all samples, the dominant OTUs were *Sphaeropsis ulmicola* (syn. *Botryodiplodia ulmicola*; 14.5% of all reads), *Gibberella baccata* (11.7%), and *Neohendersonia kickxii* (10.6%), followed by *Eutypa crustata* (4.1%), unidentified Fungi sp. (3.9%), the pathogen *O. novo-ulmi* (3.6%) and *Aureobasidium pullulans* (2.8%), *Alternaria tenuissima* (2%), and *Buckleyzyma* sp. (2%) (Supplementary Material, Table S1).

*The ~~i~~Impact of ~~h~~Host ~~i~~Identity*

Fungal community profiling across *Ulmus glabra*, *U. laevis*, and their hybrids revealed both shared and host-specific taxa. A total of 64 fungal taxa were present across all three groups, constituting a core mycobiome. *Ulmus glabra* exhibited the highest fungal richness on shoots, with 159 taxa, including 37 unique to this species. Additionally, it shared 33 taxa with *U. laevis* and 25 taxa with hybrids. *Ulmus laevis* ~~harboured~~harbored 126 taxa in total, with 20 species unique to this host and only ~~9~~nine shared exclusively with hybrids. Hybrid elms supported the lowest fungal diversity, comprising 112 taxa, including 14 unique taxa. The pairwise overlap between *U. glabra* and *U. laevis* was greater than between either species or the hybrids.

The number of taxa unique and shared among all three *Ulmus* species ~~are~~is shown in Fig. 3.

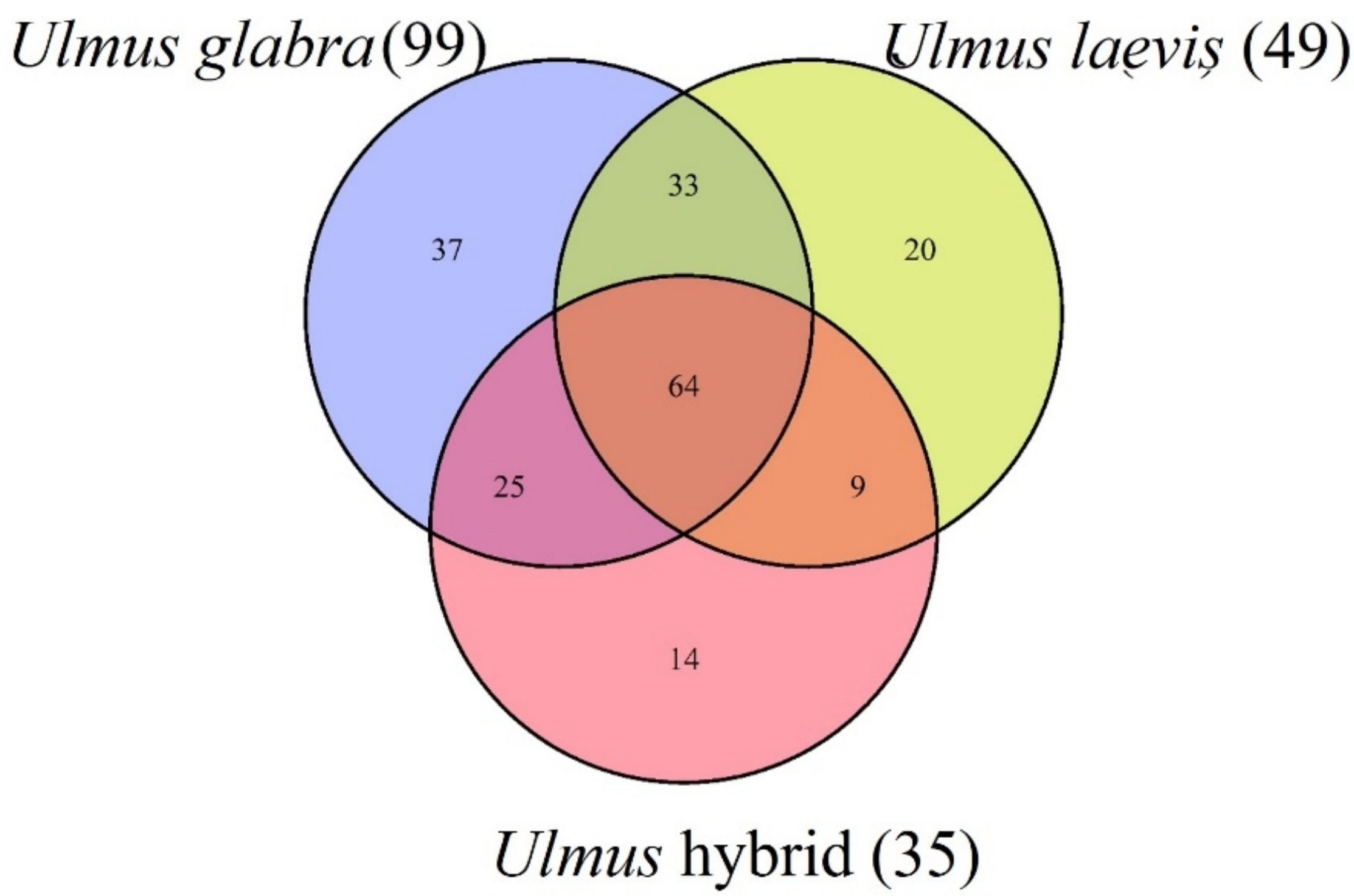


**Fig. 3**~~.~~ Venn diagram showing the number of OTUs shared and/or unique for each *Ulmus* taxa. Number in the brackets denotes the number of samples~~.~~

In *U. glabra* samples, *N. kickxii* was the most abundant species found (21.6% of all fungal sequences), followed by *S. ulmicola* (15.5%) and *G. baccata* (11.3%) (Supplementary Material, Table S2A). *Sphaeropsis ulmicola* dominated in *U. laevis* specimens (22.8% relative

abundance) (Table S2B). In the samples from hybrid elms, *G. baccata* was the dominant species, constituting over 20% of all sequences (Supplementary Material, Table S2C). *Ophiostoma novo-ulmi* was present in *U. glabra* (relative abundance 4.9%) and *U. laevis* (relative abundance 3.7%) but absent in hybrid elm samples (Supplementary Material, Table S2). Despite notable host tree-specific variation, no statistically significant differences were found among the most dominant between trees that were assessed as healthy and those that were assessed as symptomatic ($p > 0.05$) (Fig. 2, Supplementary Material, Table S2).

*The eEffect of tTree hHealth*

Rarefaction analysis showed that, on average, samples from healthy *Ulmus* trees harbouured 6.68 ± 0.78 (mean±SE) OTUs, while samples from symptomatic *Ulmus* trees harbouredharbored 11.0 ± 1.0 (mean±SE) OTUs ($F_{1,175} = 9.07$; $p < 0.05$). According to GLM analysis, species richness did not differ between the symptomatic and healthy trees (*Pp* > 0.05). The samples collected from trees in urban environments tended to have higher overall fungal species richness compared to samples from rural environments ($F_{1.183} = 1.68$; $R^2_{adj} = 0.232$; $p = 0.056$).

*Sphaeropsis ulmicola* dominated in the symptomatic trees with about 15% of all the analyszed sequences (Fig. S2) but was the fifth most common species identified in healthy trees (Supplementary Material, Table S3). *Sphaeropsis ulmicola* was detected on both symptomatic (20%) and healthy (14.5%) *U. glabra* trees, and the difference between the two health statuses werewas not statistically significant ($p = 0.19$; Fig. 4). On hybrid elms, *S. ulmicola* was present only on symptomatic trees, where it accounted for 4.1% of all sequences (Fig. 4). When viewing tree species separately, *S. ulmicola* was significantly more abundant on symptomatic *U. laevis* trees, where its sequences accounted for 24.3% of all reads, while in healthy *U. laevis* trees, this fungus accounted only for 0.14% of all reads ($p < 0.05$; Fig. 4).

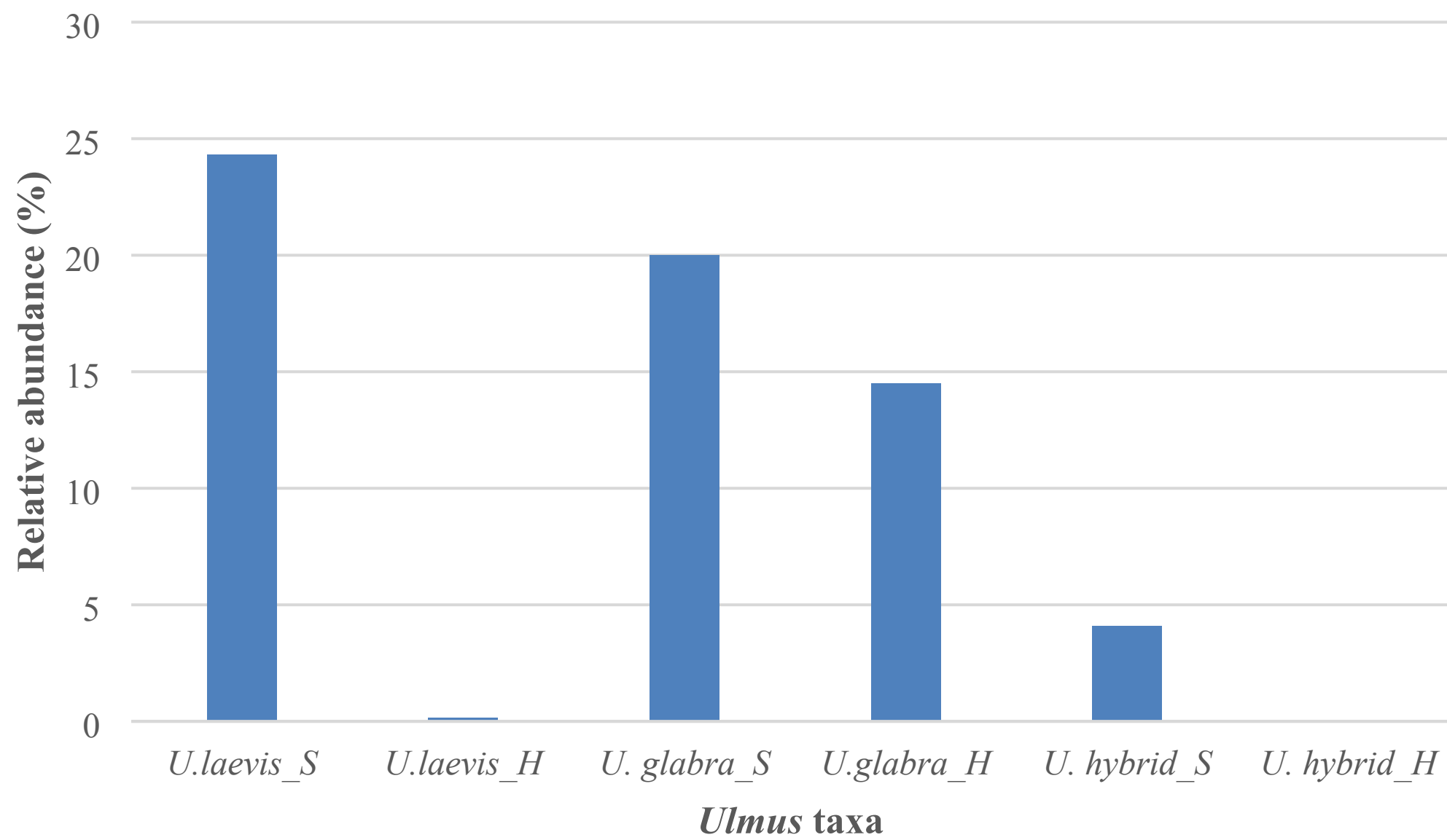


**Fig. 4**. Relative abundance of *Sphaeropsis ulmicola* on symptomatic (*S*) and healthy (*H*) *U. glabra*, *U. laevis*, and *U.* hybrids.

Samples from symptomatic trees had high abundances of *N. kickxii* and *G. baccata* sequences. In the samples from healthy trees, *G. baccata*, *Pseudophacidium ledi*, and *A. pullulans* were the three most common species (Supplementary Material, Table S3A), although none of the abovementioned species showed statistically significant differences between healthy and symptomatic trees ($p > 0.05$). *Ophiostoma novo-ulmi* was detected only in the symptomatic *U. glabra* and *U. laevis* trees (Fig. 2; Supplementary Material, Table S3B). We found no effects of *O. novo-ulmi* on the overall fungal species richness in elms. Interestingly, the presence of *S. ulmicola* was positively correlated to the overall fungal species richness ($F_{1.183} = 14.6$; $R^2_{adj} = 0.174$; $p = 0.008$). *Sphaeropsis ulmicola* and *O. novo-ulmi* were found co-occurring in only five samples across the entire dataset, all of which were from symptomatic trees.

The most common fungal taxa on symptomatic hybrid elms were *G. baccata*, *Camarosporomyces flavigenus*, and *Pleurophoma* sp. with relative abundances of 24%, 9.4%, and 5.7%, respectively. The shoots of healthy hybrid elms were dominated by *P. ledi*, *G. baccata*, and *A. tenuissima* with relative abundances of 23.6%, 23.5%, and 12.8%, respectively (Fig. 2; Supplementary Material, Table S3C).

*Differences in the ~~f~~Fungal ~~c~~Communities in ~~e~~Elm ~~t~~Trees from ~~u~~Urban vs. ~~r~~Rural ~~a~~Areas*

Twenty most abundant fungal taxa (Fig. 5~~.~~) accounted for 73.4% of all fungal reads in samples from urban environments, compared to 88.8% in rural samples of all sequences. Out of these 20 species, nine were found in both environments, whereas 11 were only found in urban spaces and ~~10~~ ten only in rural spaces. The prevalence of *O. novo-ulmi* did not differ between environments (3.2% in urban and 4.5% in rural environment; $p > 0.05$). One of the most abundant OTUs in the dataset, *E. crustata*, was found only ~~from~~in urban environments ($F_{1,184} = 7.16$; $p < 0.05$). The relative abundances of *G. baccata*, *N. kickxii*, and *S. ulmicola* did not differ significantly between the samples from urban and rural environments ($p > 0.05$). The differences in percentages of functional groups between urban and rural spaces were not statistically significant ($p > 0.05$; Supplementary Material, Table S4).

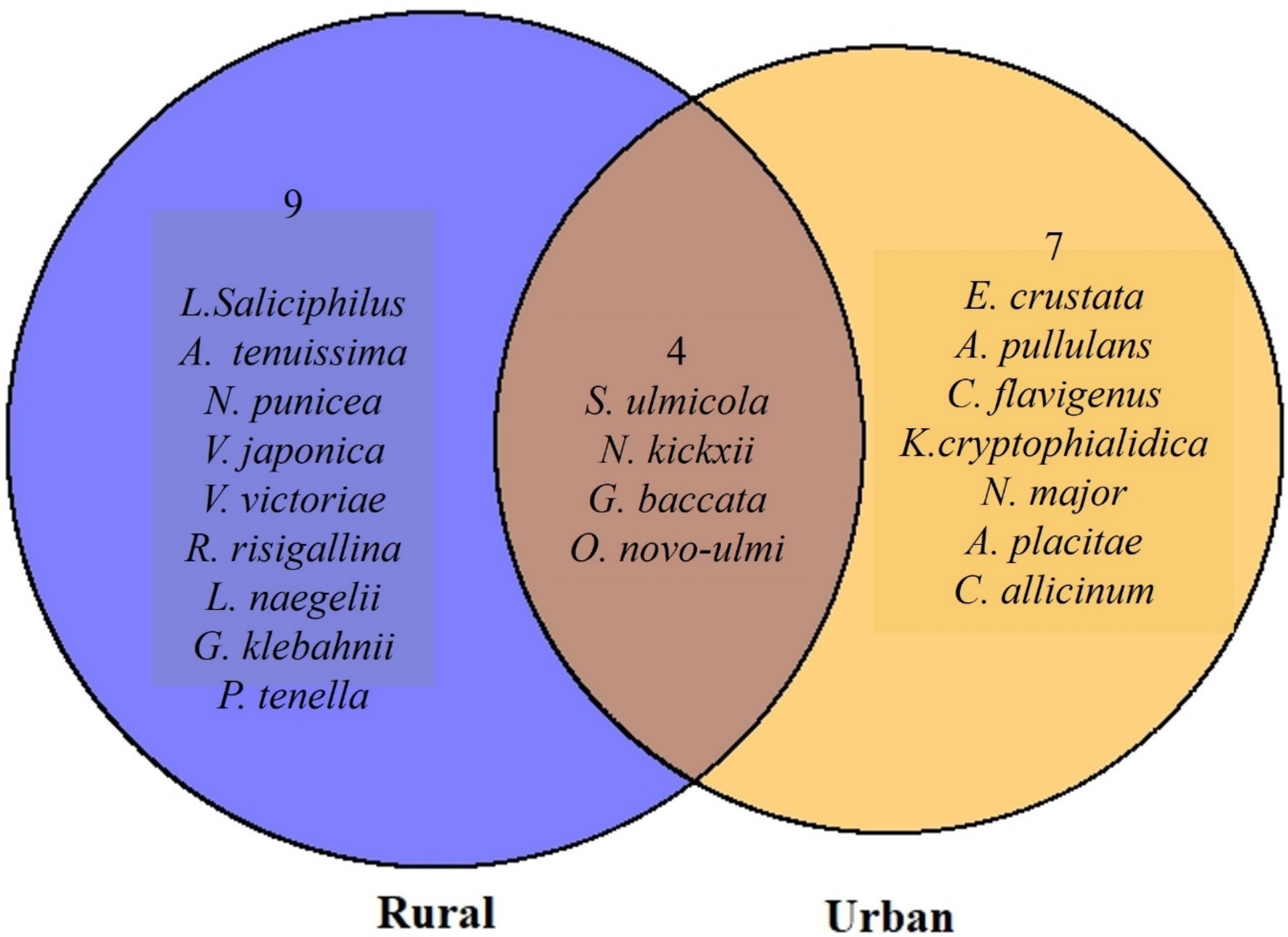


**Fig. 5**~~.~~ Venn diagram showing 20 most abundant fungal taxa that were identified to species level (unique vs shared in urban and rural environments) in healthy and symptomatic *Ulmus* trees~~.~~

*Factors ~~a~~Affecting the ~~f~~Fungal ~~c~~Community ~~c~~Composition*

Of the studied factors, fungal species composition was influenced mostly by the presence of *S. ulmicola* (PERMANOVA, 4.47% of variation explained; $p < 0.01$), followed by sampling location (Estonia vs. Russia; 2.51%; $p < 0.01$), tree species (2.32%; $p < 0.01$), and the interaction between tree species and health status (1.87%; $p < 0.05$), health status (1.86; $p < 0.01$) and presence of *O. novo-ulmi* (1.42%; $p < 0.01$) (Fig. 6).

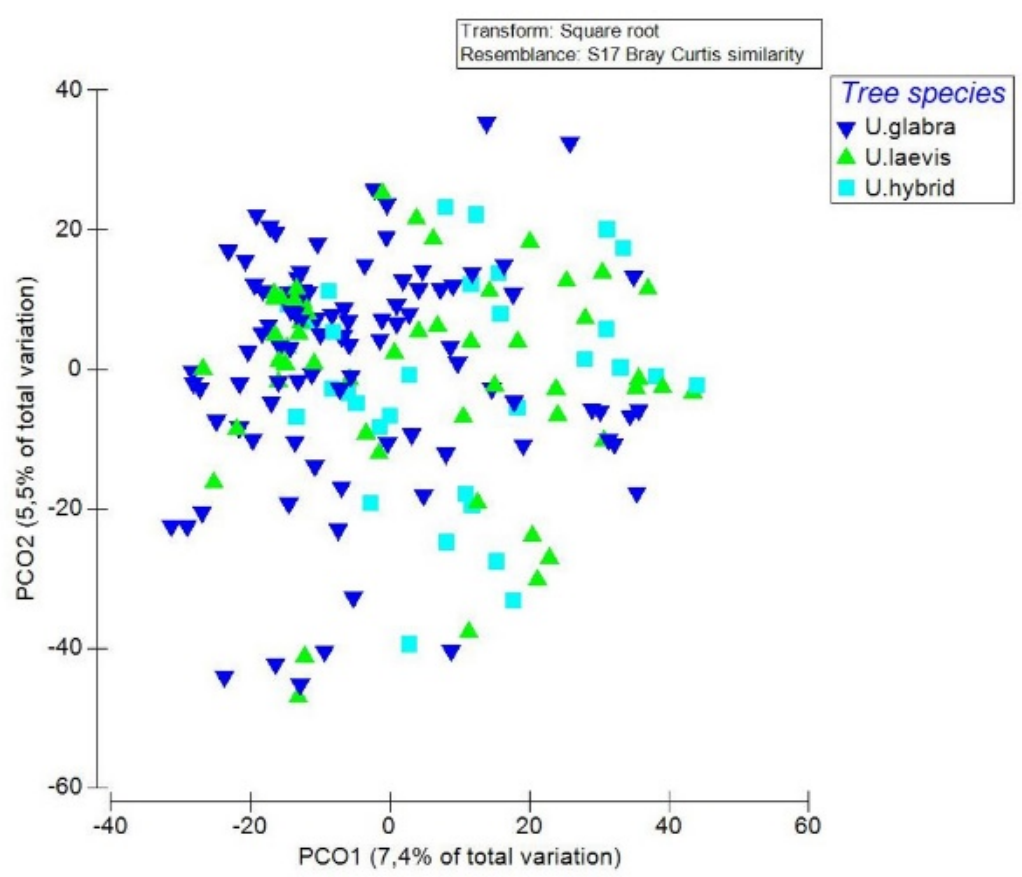


a

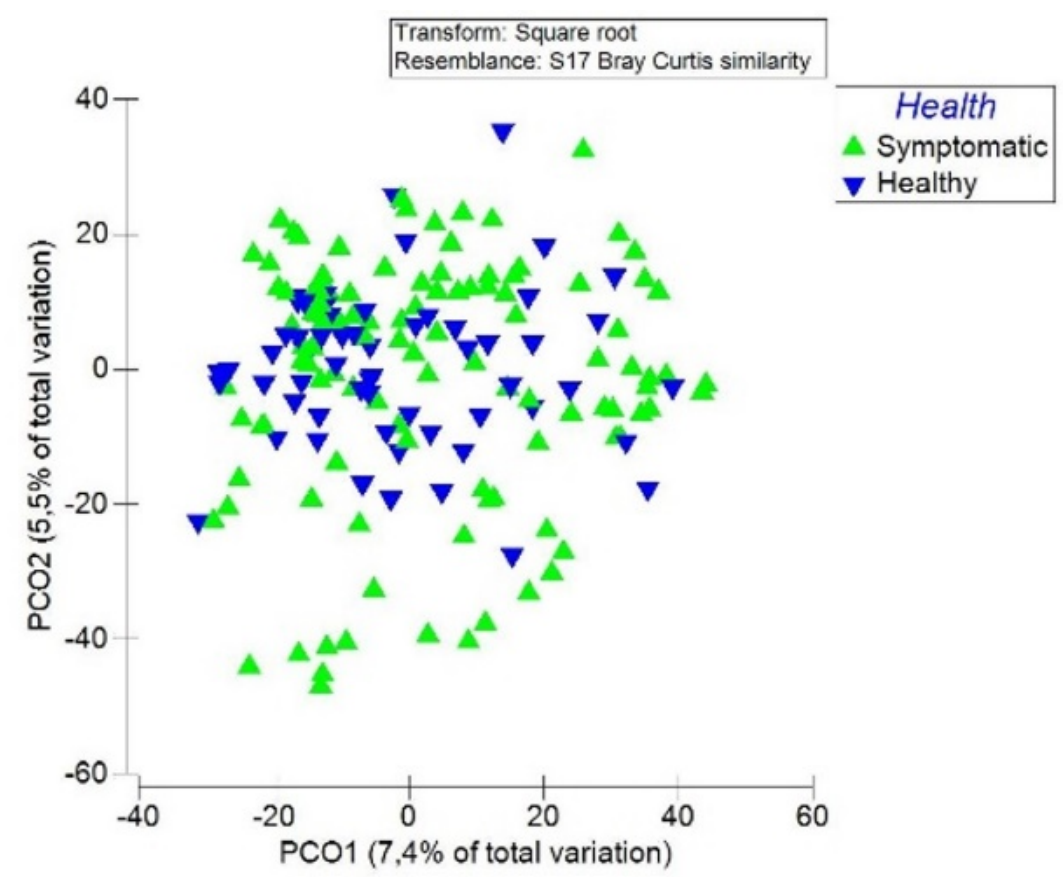


b

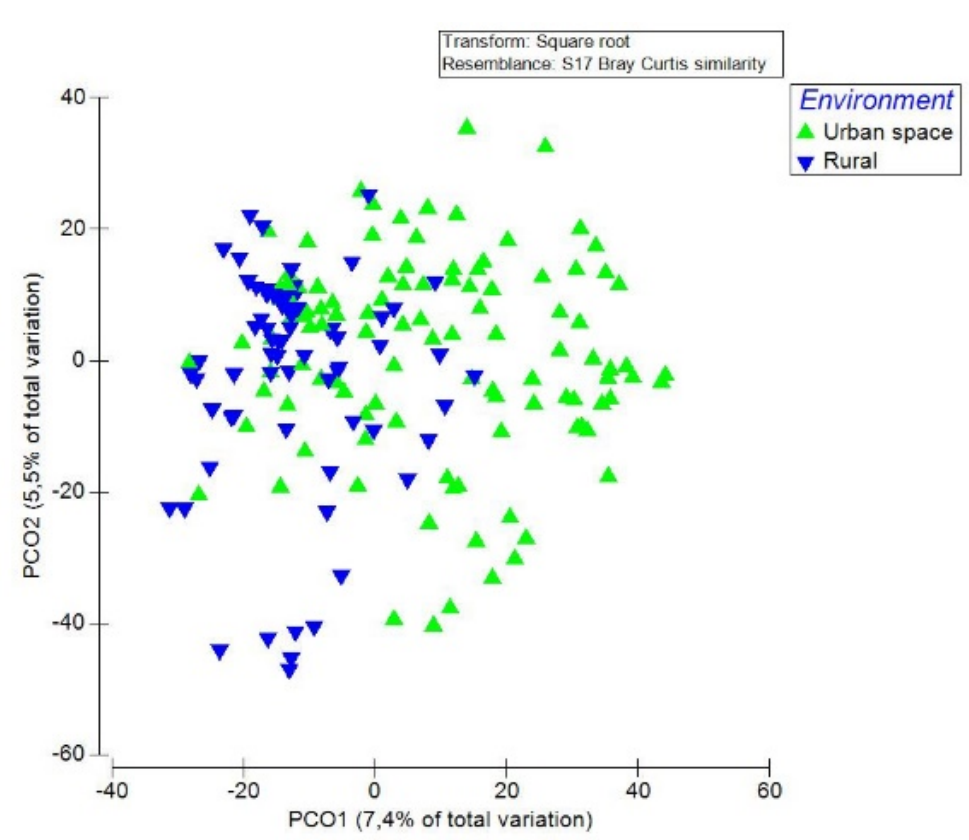


c

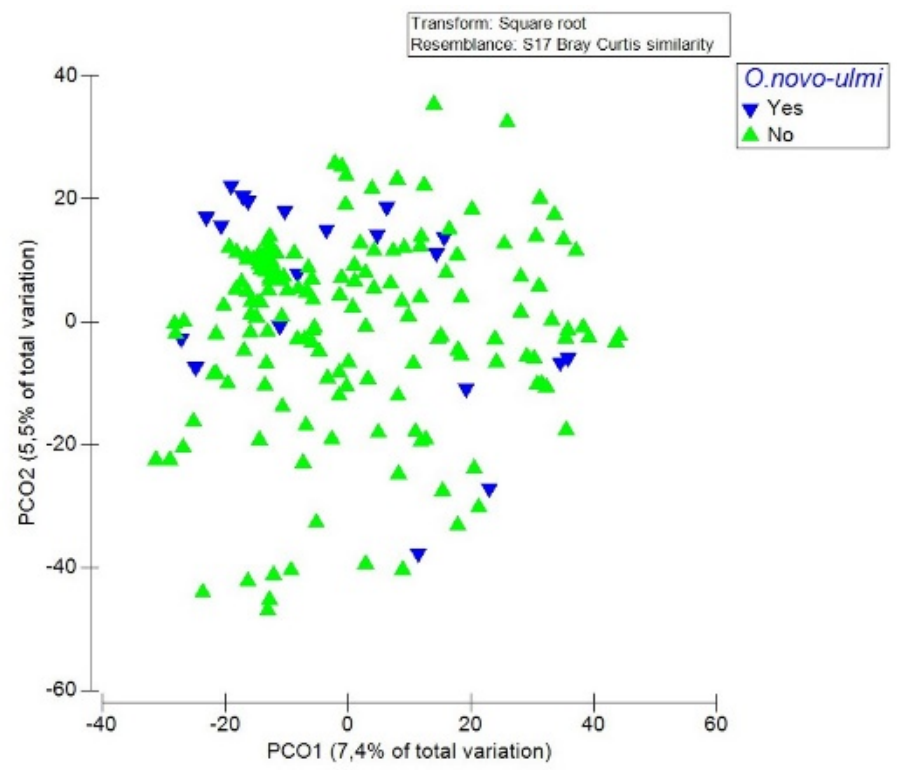


d

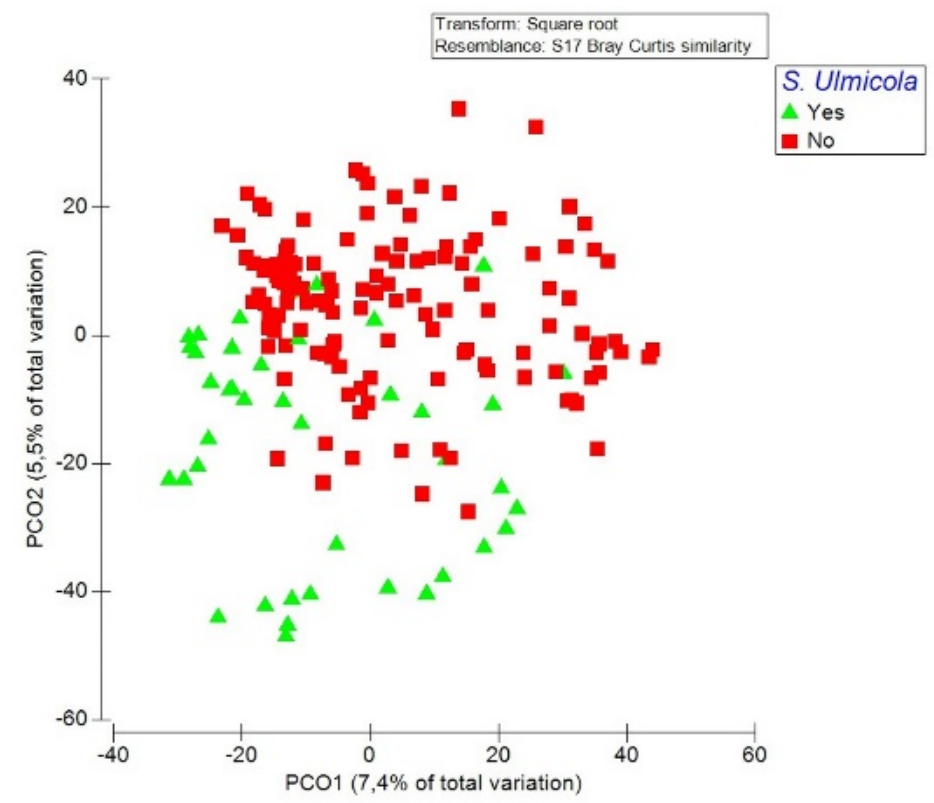


e

**Fig. 6**~~.~~ PCO plots of fungal community differences among three tree species (**a**), healthy and symptomatic trees (**b**), trees from different growth environments (**c**), trees with *O. novo-ulmi* presence/absence (**d**) and *S. ulmicola* presence/absence (**e**) according to PacBio sequencing (two main axes explain in total 12.9% of variation in the data set)~~.~~

*Species ~~i~~Interactions*

The majority of species interactions in the Estonian samples (148 of 183) were positive, while the remaining 44 were random. In contrast, random interactions dominated in the Russian samples (976 of 1164), whereas ~~the~~ 186 of 1164 were positive, and two negative associations were detected~~,~~: one between *E. crustata* and *A. pullulans* and the other between *E. crustata* and *Buckleyzyma* sp. Interactions between *O. novo-ulmi* and other fungal taxa inhabiting *Ulmus* were random (20 in the Estonian samples and 32 in the Russian samples). The OTU assigned as *Paracamarosporium fagi* showed a trend towards a negative association with *O. novo-ulmi* in the Russian samples (~~P~~*p* = 0.077).

## Discussion

This study set out to investigate how host species and phenotype influences fungal community composition in elm shoots~~—~~—a topic that remains underexplored despite its ecological and pathological significance. Understanding these host–microbe interactions is particularly relevant in the context of elm decline and attempts to conserve elms through breeding, as microbial communities can mediate disease resistance and tree health as well as climate tolerance.

The observed variation in fungal community composition among *Ulmus* taxa highlights the role of host identity in shaping fungal diversity. The elevated fungal richness in *U. glabra*, combined with its higher number of shared taxa with both *U. laevis* and hybrids, indicates a broader ecological compatibility with diverse fungal taxa, potentially due to host traits such as wood chemistry, microhabitat complexity, or evolutionary history [70]. In contrast, *U. laevis* supported fewer unique and shared taxa, indicating a more selective fungal assemblage. The markedly lower fungal richness in hybrids, coupled with minimal overlap with *U. laevis*, may reflect altered ecological interactions resulting from hybridization. This could include disrupted host traits, reduced compatibility with specialist fungi, or increased susceptibility to colonization by generalists. The consistent presence of 64 fungal taxa across all hosts suggests the existence of a stable core community, likely comprising generalist or functionally important species within *Ulmus* ecosystems [71].

Importantly, these findings indicate that *Ulmus* species are not functionally interchangeable from a mycological perspective. The significant differences in fungal diversity and community structure underscore that replacing one species with another—such as introducing hybrids or substituting *U. glabra* with *U. laevis*—could lead to a loss of host-specific fungal taxa and alter ecosystem functions mediated by these microbial communities [9]. Therefore, species-specific fungal associations should be carefully considered in conservation, restoration, and breeding programs, especially since fungal symbionts play roles in nutrient cycling, disease resistance, or resilience to environmental stress.

Investigations into fungal communities associated with elm species remain rare and are mostly based on classical microbiological methods [8, 72]. High-throughput sequencing has revolutionized our understanding of fungal diversity in forest ecosystems, revealing the richness and complexity of fungal assemblages across plant tissues and environments [5, 73]. Using this approach, we provide the first comprehensive analysis of shoot-associated fungal communities in northern European elms—including *U. glabra*, *U. laevis*, and elm hybrids—under Dutch elm disease (DED) pressure.

Our results demonstrate that elm shoots host diverse and species-rich fungal communities, similar to what has been observed in roots and leaves [8]. The consistent presence of genera

such as *Phoma*, *Sphaeropsis*, and *A. pullulans*—known saprotrophs or opportunistic pathogens [1, 5, 8, 74]—highlights elm shoots as important microhabitats and potential biodiversity reservoirs [17].

Despite some overlap, species-specific differences were evident. Surprisingly, tree species identity explained only a small fraction of the variation (~2%), unlike previous studies where host identity was a strong determinant [75]. Among the three elm taxa, elm hybrids hosted the most distinctive fungal assemblages. They were dominated by *G. baccata* (a known pathogen of *U. pumila*), *C. flavigenus*, and *Tumularia* sp.~~,~~; these species were not found in shoots of *U. glabra* or *U. laevis*, or they are rare on native hosts [58, 76-79]. This indicates that hybrid elms may support unique fungal taxa, possibly linked to their complex genetic backgrounds or stress-related vulnerability in urban sites [80, 81]. Whether these taxa are protective, neutral, or pathogenic remains unclear.

Some fungal species that have previously been reported in elm leaves and roots, such as *Trichocladium griseum* and *Penicillium restrictum* [8], were not detected in our shoot samples. This may reflect differences in fungal assemblages across plant tissues (e.g., roots, leaves, shoots), as well as the influence of tree age and seasonal variation—factors known to shape endophyte communities [9, 82-84]. These tissue-specific dynamics underscore the importance of targeted sampling when interpreting fungal diversity in woody hosts. Moreover, differences in shoot endophyte composition between native species and hybrid elms suggest that host genetic background also is an important determinant of fungal community composition [85, 86]. The genetic distinctiveness of hybrids may influence their compatibility with fungal partners, potentially contributing to the observed differences in community composition. The potential consequences of this to resistance or tolerance ~~warrants~~warrant further investigation through inoculation studies or co-culturing experiments. Interestingly, *U. laevis*— which has shown greater DED resistance [28, 87] did not display clearly different fungal compositions compared to *U. glabra*, implying that its resistance may rely more on induced defens~~c~~es or anatomical traits [9] than on endophyte-mediated protection.

Previous studies on elms and ashes have linked lower vitality and weaker defen~~e~~ses to richer fungal endophyte communities [9, 88]. In our study, symptomatic trees tended to support higher fungal species richness and ~~harboured~~harbored more stress- or decay-associated taxa. This is likely due to compromised defen~~e~~ses allowing broader fungal colonization [89, 90]. However, health status alone explained only ~2% of the variation in community composition, suggesting it is not the primary driver. These findings are consistent with earlier forest microbiome studies showing that symptomatic status alone is a poor predictor of fungal community structure unless linked with pathogen presence or environmental stressors [5, 91].

Urban trees exhibited higher fungal richness than their rural counterparts, possibly reflecting the greater species richness in urban areas, which may benefit fungal biodiversity [92]. While this contrasts with some studies reporting reduced microbial diversity in urban soils or air [93], it aligns with findings showing increased fungal colonization in stressed and species-~~richurban~~rich urban environments [94, 95]. Urbanization may lead to a shift in fungal community composition, with a decrease in symbiotrophic fungi and an increase in saprotrophic and pathogenic fungi [96]. All hybrid elms in our study were located in urban areas, and their distinct communities likely contributed to the observed richness patterns.

Environmental setting only explaining a modest portion of the variation, although it is known to play a significant modulatory role in shaping fungal communities. Similar patterns have been found in other tree species, where environmental filtering (e.g., humidity, soil, urban context) often exerts a stronger influence than host identity [1, 5, 92]. In our study, differences in fungal assemblages between elm species were less pronounced than expected, likely due to shared urban stressors such as drought, soil salinity, and pest pressure. This was particularly evident in hybrid elms, which are predominantly planted in urban areas. Geographic and management differences~~—~~—such as soil organic carbon, texture, or climatic variation~~—~~—may also have contributed to community structure, especially in the Russian samples which were all collected from urban sites [92, 97]. These results reinforce earlier findings that environmental variables can modulate or even override host-driven community patterns [98]~~,~~ and highlight that urban conditions may compromise hybrid elm health through combined abiotic and biotic stressors [28, 94, 99].

Molecularly detected pathogens, including *O. novo-ulmi* and *S. ulmicola*, explained more variation in fungal communities than visually assessed health status. *Sphaeropsis ulmicola* was frequently found in symptomatic trees that tested negative for *O. novo-ulmi*, consistent with studies in Estonia and Poland [28, 38]. While DED has previously been associated with reduced fungal diversity [8], in our study, the presence of *O. novo-ulmi* did not significantly alter fungal community composition. This pattern suggests a possible ecological separation or exclusion between the two pathogens. *Sphaeropsis ulmicola* stood out as a potentially underestimated pathogen in northern elm populations. *Sphaeropsis ulmicola* is ~~nown~~known to cause symptoms similar to DED and has previously been associated with canker disease in *Ulmus* spp. [38, 100]. In our study, it was highly prevalent in symptomatic but *O. novo-ulmi*-negative trees (see Supplementary Table S4), reflecting patterns observed in Estonia where co-occurrence of the two pathogens is rare [28].

Interestingly, trees harbouring *S. ulmicola* exhibited higher overall fungal richness, which may reflect its role in weakening host defen~~e~~ses or altering tissue structure to facilitate further colonization. While this co-occurrence does not confirm interaction, it merits further functional investigation.

The dual role of *S. ulmicola* as both a latent endophyte and opportunistic pathogen is intriguing. It was detected in both symptomatic (20%) and asymptomatic (14.5%) *U. glabra* trees, suggesting that it may behave similarly to *Diplodia sapinea* in pines: typically quiescent but capable of causing disease under stress, such as drought [101, 102]. Its absence in healthy hybrid elms and minimal presence in healthy *U. laevis* indicate that in these taxa, infection likely occurs through secondary colonization (e.g., airborne spores) during host stress rather than through latent residency. Further research is needed to clarify *S. ulmicola*~~'s~~’s infection strategy in Nordic environments.

In hybrid elms, the most frequent fungi were *G. baccata* (*Fusarium lateritium*), *C. flavigenus*, and *Tumularia* sp. In earlier studies, *G. baccata* has been linked to disease outbreaks in *U. pumila* [78], and *C. flavigenus* is a ~~p~~*Phoma*-like fungus [73, 79], based on morphological features, does not reflect natural evolutionary history of this group of fungi [103]. These findings

suggest that hybrid elms may be challenged by both biotic and abiotic stress, particularly if hybrid elms are lacking genetic capacity to tolerate cold or drought [80, 81].

Overall, fungal community dynamics in elm shoots appear shaped by a complex interplay of stress, site conditions, host genetics, and disease agents. Environmental stressors and urban context may override host-specific effects, particularly in hybrids. These findings emphasize the need for integrated approaches combining molecular diagnostics, ecological data, and functional studies to understand elm–fungus interactions in changing environments.

## Conclusions

Our study demonstrates that fungal community dynamics in elm shoots are shaped by a complex interplay of factors, with environmental conditions~~—~~—particularly the contrast urban versus rural settings~~—~~—and pathogen presence emerging as stronger drivers than host species identity or visual health status. While species-rich fungal assemblages were detected across all elm taxa, host-specific effects on community composition were modest. Hybrid elms planted exclusively in urban environments hosted distinct fungal communities, likely reflecting both their genetic background and the influence of urban stressors. *Ulmus glabra* exhibited the highest fungal richness on shoots, which indicates that *Ulmus* species are not replaceable from a microfungal perspective.

Symptomatic trees, especially those ~~with~~ harbo~~u~~ring pathogens, exhibited consistently higher fungal richness, supporting the notion that weakened host defen~~c~~ses enable broader fungal colonization. However, health status alone explained only a small portion of the variation in fungal community composition. *Sphaeropsis ulmicola* was frequently detected in symptomatic but *Ophiostoma novo-ulmi*-negative trees, raising the possibility that it acts as an emerging pathogen in northern elm populations, including in Dutch elm disease–resistant hosts~~-~~. These findings underscore the multifactorial nature of fungal community assembly in elms and highlight the need for integrated approaches that consider host genotype, pathogen dynamics, tree health, and site context. Future research should include functional studies on key fungal taxa to better understand their roles in host resilience and disease progression.

**Supplementary ~~material~~Information**

Table S1. Abundance of all fungal species present in *Ulmus* taxa that had a relative abundance > 0.1 percent of all sequences

| Species | Percentage |
|---|---|
| Sphaeropsis ulmicola | 14.46 |
| Gibberella baccata | 11.68 |
| Neohendersonia kickxii | 10.58 |
| Eutypa crustata | 4.13 |
| Fungi sp | 3.99 |
| Ophiostoma novo-ulmi | 3.61 |
| Cladosporium sp | 2.82 |
| Aureobasidium pullulans | 2.75 |
| Fungi sp. | 2.21 |
| Alternaria tenuissima | 2.03 |
| Buckleyzyma sp | 2.01 |
| Cadophora sp | 1.75 |
| Pleurophoma sp | 1.69 |
| Camarosporomyces flavigenus | 1.69 |
| Knufia cryptophialidica | 1.65 |
| Tumularia sp | 1.58 |
| Liberomyces saliciphilus | 1.55 |
| Labyrinthomyces sp | 1.40 |
| Pseudophacidium ledi | 1.12 |
| Antennariella placitae | 1.08 |
| Neonectria major | 0.94 |
| Phoma sp | 0.86 |
| Vishniacozyma victoriae | 0.87 |
| Neosetophoma sp | 0.85 |
| Helotiales sp | 0.80 |
| Cladosporium allicinum | 0.72 |
| Hypocreales sp | 0.72 |
| Ascomycota sp | 0.68 |
| Winterella hypodermia | 0.64 |
| Chaetothyriales sp | 0.58 |
| Neonectria punicea | 0.45 |
| Teratosphaeriaceae sp | 0.44 |
| Aspergillus sp | 0.43 |
| Pyrenochaeta sp | 0.41 |

| Species | Percentage |
| --- | --- |
| Fungi sp | 0.38 |
| Didymella heteroderae | 0.36 |
| Pestalotiopsis sp | 0.37 |
| Myriangiales sp | 0.36 |
| Muriformistrickeria rosae | 0.33 |
| Phaeosphaeriopsis sp | 0.32 |
| Capnodium citri | 0.29 |
| Paracamarosporium fagi | 0.28 |
| Vishniacozyma carnescens | 0.27 |
| Cucurbitariaceae sp | 0.27 |
| Cladophialophora sp | 0.27 |
| Fungi sp | 0.27 |
| Fungi sp | 0.26 |
| Chaetothyriales sp | 0.26 |
| Dothideales sp | 0.26 |
| Alatosessilispora bibrachiata | 0.25 |
| Filobasidium sp | 0.25 |
| Phaeosphaeria sp | 0.25 |
| Veronaea japonica | 0.24 |
| Reddellomyces sp | 0.24 |
| Physcia tenella | 0.22 |
| Helotiales sp | 0.22 |
| Russula risigallina | 0.22 |
| Vishniacozyma sp | 0.19 |
| Pleosporales sp | 0.19 |
| Fungi sp | 0.18 |
| Lecania naegelii | 0.18 |
| Coprinellus disseminatus | 0.18 |
| Taphrina ulmi | 0.17 |
| Phaeophyscia orbicularis | 0.17 |
| Geotrichum klebahnii | 0.16 |
| Cucurbitariaceae sp | 0.15 |
| Fibulobasidium inconspicuum | 0.15 |
| Pseudeurotiaceae sp | 0.15 |
| Monocillium nordinii | 0.15 |
| Fungi sp | 0.14 |
| Fungi sp | 0.14 |
| Capronia sp | 0.13 |
| Phomatodes sp | 0.12 |
| Phaeothecoidea proteae | 0.11 |

| Species | Percentage |
|---|---|
| Herpotrichiellaceae sp | 0.11 |
| Pleosporales sp | 0.11 |
| Orbiliales sp | 0.10 |
| Paratritirachium sp | 0.10 |
| Trichomeriaceae sp | 0.09 |

Table S2. Abundance and functional group of 20 most abundant fungal OTU-s detected on *U. glabra* (a), *U. laevis* (b) and hybrid elms (c).

a

| **Species** | **Percentage** | **Functional group** |
|---|---|---|
| *Neohendersonia kickxii* | 21.65 | Plant endophyte |
| *Sphaeropsis ulmicola* | 15.55 | Plant pathogen |
| *Gibberella baccata* | 11.27 | Undefined saprotroph |
| *Eutypa crostata* | 7.91 | Wood saprotroph |
| *Ophiostoma novo-ulmi* | 4.94 | Plant pathogen |
| *Labyrinthomyces sp* | 2.88 | Ectomycorrhizal |
| *Antennariella placitae* | 2.19 | Undefined saprotroph |
| *Fungi sp2* | 2.06 | - |
| *Cladosporium sp* | 2.01 | Mold |
| *Knufia cryptophialidica* | 1.97 | Plant epiphyte |
| *Alternaria tenuissima* | 1.86 | Plant endophyte |
| *Hypocreales sp* | 1.48 | - |
| *Cadophora sp* | 1.29 | Plant pathogen |
| *Aureobasidium pullulans* | 1.08 | Plant endophyte |
| *Fungi sp1* | 0.90 | - |
| *Aspergillus sp* | 0.90 | Undefined saprotroph |
| *Vishniacozyma victoriae* | 0.84 | Yeast |
| *Helotiales sp* | 0.76 | - |
| *Chaetothyriales sp* | 0.71 | - |
| *Phaeosphaeriopsis sp* | 0.64 | Plant pathogen |

b

| **Species** | **Percentage** | **Functional group** |
|---|---|---|
| *Sphaeropsis ulmicola* | 22.83 | Plant pathogen |
| *Fungi sp1* | 11.76 | - |
| *Liberomyces saliciphilus* | 5.54 | Plant pathogen |
| *Buckleyzyma sp* | 5.51 | Yeast |
| *Cladosporium sp* | 3.83 | Mold |

| | | |
|---|---|---|
| *Ophiostoma novo-ulmi* | 3.73 | Plant pathogen |
| *Gibberella baccata* | 2.77 | Undefined saprotroph |
| *Fungi sp2* | 2.64 | - |
| *Pleurophoma sp* | 2.49 | Plant pathogen |
| *Aureobasidium pullulans* | 2.47 | Plant endophyte |
| *Winterella hypodermia* | 2.42 | Mold |
| *Knufia cryptophialidica* | 2.37 | Plant epiphyte |
| *Cadophora sp* | 2.04 | Plant pathogen |
| *Neonectria punicea* | 1.69 | Undefined saprotroph |
| *Helotiales sp* | 1.49 | - |
| *Pestalotiopsis sp* | 1.38 | Plant pathogen |
| *Phoma sp* | 1.21 | Plant pathogen |
| *Eutypa crustata* | 1.01 | Wood saprotroph |
| *Vishniacozyma victoriae* | 1.01 | Yeast |
| *Paracamarosporium fagi* | 0.97 | Yeast |

c

| **Species** | **Percentage** | **Functional group** |
|---|---|---|
| *Gibberella baccata* | 22.25 | Undefined saprotroph |
| *Camarosporomyces flavigenus* | 6.92 | - |
| *Tumularia sp* | 6.45 | Plant pathogen |
| *Aureobasidium pullulans* | 6.34 | Plant endophyte |
| *Pseudophacidium ledi* | 4.59 | Wood saprotroph |
| *Pleurophoma sp* | 4.21 | Plant pathogen |
| *Neonectria major* | 3.87 | Undefined saprotroph |
| *Alternaria tenuissima* | 3.71 | Plant endophyte |
| *Cladosporium sp* | 3.35 | Mold |
| *Neosetophoma sp* | 3.10 | Plant saprotroph |
| *Sphaeropsis ulmicola* | 3.02 | Plant pathogen |
| *Cladosporium allicinum* | 2.63 | Mold |
| *Ascomycota sp* | 2.46 | - |
| *Cadophora sp* | 2.30 | Plant pathogen |
| *Fungi sp2* | 2.05 | - |
| *Fungi sp1* | 1.61 | - |
| *Phoma sp* | 1.61 | Plant pathogen |
| *Pyrenochaeta sp* | 1.61 | Plant pathogen |
| *Myriangiales sp* | 1.44 | Plant pathogen |
| *Buckleyzyma sp* | 1.36 | Yeast |

Table S3. Abundance and functional group of 20 most abundant fungal OTU-s detected on healthy (a) and symptomatic (b) trees in the dataset (*U. glabra*, *U. laevis* and hybrid elms combined).

a

| **Species** | **Percentage** | **Functional group** |
|---|---|---|
| *Gibberella baccata* | 9.16 | General saprotroph |
| *Pseudophacidium ledi* | 8.59 | Wood saprotroph |
| *Aureobasidium pullulans* | 7.09 | Plant endophyte |
| *Cladosporium sp* | 6.78 | Mold |
| *Sphaeropsis ulmicola* | 5.33 | Plant pathogen |
| *Alternaria tenuissima* | 5.12 | Plant endophyte |
| *Pleurophoma sp* | 5.12 | Plant pathogen |
| *Winterella hypodermia* | 4.97 | Mold |
| *Pestalotiopsis sp* | 2.85 | Plant pathogen |
| *Phoma sp* | 2.80 | Plant pathogen |
| *Paracamarosporium fagi* | 1.97 | Yeast |
| *Antennariella placitae* | 1.86 | General saprotroph |
| *Vishniacozyma victoriae* | 1.76 | Yeast |
| *Cladosporium allicinum* | 1.71 | Mold |
| *Muriformistrickeria rosae* | 1.60 | - |
| *Fungi sp.* | 1.40 | - |
| *Teratosphaeriaceae sp* | 1.35 | - |
| *Buckleyzyma sp* | 1.24 | Yeast |
| *Fibulobasidium inconspicuum* | 1.14 | - |
| *Didymella heteroderae* | 0.98 | Plant pathogen |

b

| **Species** | **Percentage** | **Functional group** |
|---|---|---|
| *Sphaeropsis ulmicola* | 15.12 | Plant pathogen |
| *Neohendersonia kickxii* | 13.26 | Plant endophyte |
| *Gibberella baccata* | 13.16 | General saprotroph |
| *Eutypa crustata* | 5.17 | Wood saprotroph |
| *Fungi sp1* | 4.87 | - |
| *Ophiostoma novo-ulmi* | 4.52 | Plant pathogen |
| *Fungi sp2* | 2.47 | - |
| *Cladosporium sp* | 2.38 | Mold |
| *Buckleyzyma sp* | 2.29 | Yeast |
| *Aureobasidium pullulans* | 2.28 | Plant endophyte |
| *Cadophora sp* | 2.21 | Plant pathogen |
| *Camarosporomyces flavigenus* | 2.12 | - |
| *Knufia cryptophialidica* | 1.91 | Plant epiphyte |
| *Alternaria tenuissima* | 1.64 | Plant endophyte |
| *Pleurophoma sp* | 1.29 | Plant pathogen |
| *Neonectria major* | 1.19 | General saprotroph |
| *Antennariella placitae* | 1.05 | General saprotroph |

| | | |
|---|---|---|
| *Neosetophoma sp* | 1.01 | Plant saprotroph |
| *Helotiales sp* | 0.96 | - |
| *Hypocreales sp* | 0.91 | - |

Table S4. The differences in percentages of most abundant taxa between urban and rural spaces.

| **Rural space** | | **Urban space** | |
|---|---|---|---|
| **Taxon** | **Percentage** | **Taxon** | **Percentage** |
| *Sphaeropsis ulmicola* | 31.16 | *Gibberella baccata* | 9.97 |
| *Neohendersonia kickxii* | 20.70 | *Eutypa crustata* | 7.89 |
| *Gibberella baccata* | 16.83 | *Neohendersonia kickxii* | 7.78 |
| *Ophiostoma novo-ulmi* | 4.95 | Fungi sp. 1 | 7.44 |
| *Liberomyces saliciphilus* | 4.86 | *Sphaeropsis ulmicola* | 7.06 |
| *Alternaria tenuissima* | 3.10 | *Ophiostoma novo-ulmi* | 3.91 |
| *Hypocreales* sp. | 2.26 | Fungi sp. 2 | 3.73 |
| *Neonectria punicea* | 1.44 | *Buckleyzyma* sp. | 3.50 |
| *Aspergillus* sp. | 1.31 | *Aureobasidium pullulans* | 3.47 |
| *Phaeosphaeriopsis* sp. | 0.95 | *Cladosporium* sp. | 3.46 |
| *Cadophora* sp. | 0.77 | *Camarosporomyces flavigenus* | 3.24 |
| *Veronaea japonica* | 0.71 | *Knufia cryptophialidica* | 2.92 |
| *Vishniacozyma victoriae* | 0.67 | *Cadophora* sp. | 2.90 |
| Fungi sp. | 0.52 | *Pleurophoma* sp. | 1.97 |
| *Russula risigallina* | 0.45 | *Neonectria major* | 1.81 |
| *Lecania naegelii* | 0.45 | *Antennariella placitae* | 1.57 |
| *Geotrichum klebahnii* | 0.41 | *Neosetophoma* sp. | 1.49 |
| *Cladosporium* sp. | 0.39 | *Helotiales* sp. | 1.44 |
| *Teratosphaeriaceae* sp. | 0.39 | *Ascomycota* sp. | 1.31 |
| *Physcia tenella* | 0.39 | *Cladosporium allicinum* | 0.93 |

Table S5. Data of samples.

| | Country | Name of the site | Coordinates | Environment | Habitat | Host | Sampling date | Canopy dieback | ID No |
|---|---|---|---|---|---|---|---|---|---|
| 1 | Estonia | Tihemetsa | 58.147583, 25.039967 | rural | park | *U. glabra* | 10.07.2016 | yes | **6377** |
| 2 | Estonia | Tihemetsa | 58.147600,25.040633 | rural | park | *U. glabra* | 10.07.2016 | yes | **6378** |
| 3 | Estonia | Tihemetsa | 58.148417,25.046700 | rural | park | *U. glabra* | 10.07.2016 | yes | **6392** |
| 4 | Estonia | Tihemetsa | 58.148383,25.046783 | rural | park | *U. glabra* | 10.07.2016 | yes | **6394** |
| 5 | Estonia | Tihemetsa | 58.148433,25.046983 | rural | park | *U. glabra* | 10.07.2016 | yes | **6395** |
| 6 | Estonia | Tihemetsa | 58.148033,25.042817 | rural | park | *U. glabra* | 10.07.2016 | yes | **6374** |
| 7 | Estonia | Tihemetsa | 58.149517,25.047600 | rural | park | *U. glabra* | 10.07.2016 | yes | **6400** |
| 8 | Estonia | Tihemetsa | 58.149550,25.047433 | rural | park | *U. glabra* | 10.07.2016 | yes | **6401** |
| 9 | Estonia | Tihemetsa | 58.149033,25.044867 | rural | park | *U. glabra* | 10.07.2016 | yes | **6383** |
| 10 | Estonia | Tihemetsa | 58.148083,25.043533 | rural | park | *U. glabra* | 10.07.2016 | yes | **6373** |
| 11 | Estonia | Tihemetsa | 58.149033,25.044583 | rural | park | *U. glabra* | 10.07.2016 | yes | **6384** |
| 12 | Estonia | Tihemetsa | 58.149233,25.044817 | rural | park | *U. glabra* | 10.07.2016 | yes | **6387** |
| 13 | Estonia | Tihemetsa | 58.149567,25.045500 | rural | park | *U. glabra* | 10.07.2016 | yes | **6389** |
| 14 | Estonia | Tihemetsa | 58.149517,25.045650 | rural | park | *U. glabra* | 10.07.2016 | yes | **8155** |
| 15 | Estonia | Tihemetsa | 58.149533,25.045683 | rural | park | *U. glabra* | 10.07.2016 | yes | **6391** |
| 16 | Estonia | Tihemetsa | 58.148383,25.046783 | rural | park | *U. glabra* | 10.07.2016 | yes | **6393** |
| 17 | Estonia | Tihemetsa | 58.148383,25.046967 | rural | park | *U. glabra* | 10.07.2016 | yes | **6396** |
| 18 | Estonia | Tihemetsa | 58.148483,25.042067 | rural | park | *U. glabra* | 10.07.2016 | no | **8139** |
| 19 | Estonia | Tihemetsa | 58.147850,25.039867 | rural | park | *U. glabra* | 10.07.2016 | no | **8140** |
| 20 | Estonia | Tihemetsa | 58.147650,25.039517 | rural | park | *U. glabra* | 10.07.2016 | no | **8142** |
| 21 | Estonia | Tihemetsa | 58.147650,25.039350 | rural | park | *U. glabra* | 10.07.2016 | no | **8143** |
| 22 | Estonia | Tihemetsa | 58.147583,25.039767 | rural | park | *U. glabra* | 10.07.2016 | no | **8145** |
| 23 | Estonia | Tihemetsa | 58.149383,25.044983 | rural | park | *U. glabra* | 10.07.2016 | no | **8149** |
| 24 | Estonia | Tihemetsa | 58.149533,25.044933 | rural | park | *U. glabra* | 10.07.2016 | no | **8150** |
| 25 | Estonia | Tihemetsa | 58.149633,25.045400 | rural | park | *U. glabra* | 10.07.2016 | no | **8152** |
| 26 | Estonia | Tihemetsa | 58.149667,25.047500 | rural | park | *U. glabra* | 10.07.2016 | no | **8191** |

| | Country | Name of the site | Coordinates | Environment | Habitat | Host | Sampling date | Canopy dieback | ID No |
|---|---|---|---|---|---|---|---|---|---|
| 27 | Estonia | Tallinn | 59.426433, 24.776550 | urban space | street | *U. glabra* | 14.07.2016 | yes | **6446** |
| 28 | Estonia | Tallinn | 59.426317,24.776600 | urban space | street | *U. glabra* | 14.07.2016 | yes | **6447** |
| 29 | Estonia | Tallinn | 59.427950,24.720317 | urban space | street | *U. glabra* | 14.07.2016 | yes | **6450** |
| 30 | Estonia | Tallinn | 59.446783,24.689267 | urban space | park | *U. glabra* | 14.07.2016 | yes | **6452** |
| 31 | Estonia | Tallinn | 59.451467,24.690700 | urban space | street | *U. glabra* | 14.07.2016 | yes | **6453** |
| 32 | Estonia | Tallinn | 59.452133,24.692500 | urban space | street | *U. glabra* | 14.07.2016 | yes | **6456** |
| 33 | Estonia | Tallinn | 59.451883,24.691733 | urban space | street | *U. glabra* | 14.07.2016 | yes | **6459** |
| 34 | Estonia | Tallinn | 59.451650,24.685083 | urban space | park | *U. glabra* | 14.07.2016 | yes | **6501** |
| 35 | Estonia | Tallinn | 59.425283,24.775683 | urban space | street | *U. glabra* | 14.07.2016 | yes | **6448** |
| 36 | Estonia | Tallinn | 59.451583,24.691117 | urban space | street | *U. glabra* | 14.07.2016 | yes | **6455** |
| 37 | Estonia | Tallinn | 59.451583,24.691117 | urban space | street | *U. glabra* | 14.07.2016 | yes | **6457** |
| 38 | Estonia | Tallinn | 59.449350,24.684417 | urban space | park | *U. glabra* | 14.07.2016 | yes | **6460** |
| 39 | Estonia | Tallinn | 59.449350,24.684417 | urban space | park | *U. glabra* | 14.07.2016 | yes | **6462** |
| 40 | Estonia | Tallinn | 59.451650,24.685083 | urban space | park | *U. glabra* | 14.07.2016 | yes | **6500** |

| | Country | Name of the site | Coordinates | Environment | Habitat | Host | Sampling date | Canopy dieback | ID No |
|---|---|---|---|---|---|---|---|---|---|
| 41 | Estonia | Tallinn | 59.425900,24.776650 | urban space | street | *U. glabra* | 14.07.2016 | yes | **8348** |
| 42 | Estonia | Tallinn | 59.452850,24.683867 | urban space | park | *U. glabra* | 14.07.2016 | yes | **8359** |
| 43 | Estonia | Tallinn | 59.451583,24.691117 | urban space | street | *U. glabra* | 14.07.2016 | no | **8351** |
| 44 | Estonia | Tallinn | 59.452167,24.692800 | urban space | street | *U. glabra* | 14.07.2016 | no | **8353** |
| 45 | Estonia | Tallinn | 59.452350,24.692767 | urban space | street | *U. glabra* | 14.07.2016 | no | **8354** |
| 46 | Estonia | Tallinn | 59.452233,24.692667 | urban space | street | *U. glabra* | 14.07.2016 | no | **8355** |
| 47 | Estonia | Tallinn | 59.452417,24.684233 | urban space | park | *U. glabra* | 14.07.2016 | no | **8356** |
| 48 | Estonia | Tallinn | 59.452300,24.684167 | urban space | park | *U. glabra* | 14.07.2016 | no | **8357** |
| 49 | Estonia | Tallinn | 59.452567,24.682667 | urban space | park | *U. glabra* | 14.07.2016 | no | **8358** |
| 50 | Estonia | Tallinn | 59.454133,24.682700 | urban space | park | *U. glabra* | 14.07.2016 | no | **8362** |
| 51 | Estonia | Tallinn | 59.454533,24.683583 | urban space | park | *U. glabra* | 14.07.2016 | no | **8363** |
| 52 | Estonia | Heimtali | 58.333917,25.510783 | rural | forest | *U. glabra* | 20.07.2016 | yes | **6553** |
| 53 | Estonia | Heimtali | 58.333867,25.510400 | rural | forest | *U. glabra* | 20.07.2016 | yes | **6554** |
| 54 | Estonia | Heimtali | 58.333717,25.509500 | rural | forest | *U. glabra* | 20.07.2016 | yes | **6555** |
| 55 | Estonia | Heimtali | 58.323900,25.536133 | rural | forest | *U. glabra* | 20.07.2016 | yes | **6564** |
| 56 | Estonia | Heimtali | 58.323683,25.536417 | rural | forest | *U. glabra* | 20.07.2016 | yes | **6568** |
| 57 | Estonia | Heimtali | 58.323683,25.536417 | rural | forest | *U. glabra* | 20.07.2016 | yes | **6700** |

| | Country | Name of the site | Coordinates | Environment | Habitat | Host | Sampling date | Canopy dieback | ID No |
|---|---|---|---|---|---|---|---|---|---|
| 58 | Estonia | Heimtali | 58.323350,25.537133 | rural | forest | *U. glabra* | 20.07.2016 | yes | **6699** |
| 59 | Estonia | Heimtali | 58.323350,25.537133 | rural | forest | *U. glabra* | 20.07.2016 | yes | **6701** |
| 60 | Estonia | Heimtali | 58.323350,25.537133 | rural | forest | *U. glabra* | 20.07.2016 | yes | **6702** |
| 61 | Estonia | Heimtali | 58.334500,25.511050 | rural | forest | *U. glabra* | 20.07.2016 | yes | **6550** |
| 62 | Estonia | Heimtali | 58.333017,25.507283 | rural | forest | *U. glabra* | 20.07.2016 | yes | **6556** |
| 63 | Estonia | Heimtali | 58.332200,25.521483 | rural | forest | *U. glabra* | 20.07.2016 | yes | **6558** |
| 64 | Estonia | Heimtali | 58.329967,25.526300 | rural | forest | *U. glabra* | 20.07.2016 | yes | **6563** |
| 65 | Estonia | Heimtali | 58.329967,25.526300 | rural | forest | *U. glabra* | 20.07.2016 | yes | **6565** |
| 66 | Estonia | Heimtali | 58.323900,25.536133 | rural | forest | *U. glabra* | 20.07.2016 | yes | **6566** |
| 67 | Estonia | Heimtali | 58.333100,25.523533 | rural | forest | *U. glabra* | 20.07.2016 | yes | **8310** |
| 68 | Estonia | Heimtali | 58.333100,25.524067 | rural | forest | *U. glabra* | 20.07.2016 | yes | **8313** |
| 69 | Estonia | Poruni | 59.174528, 27.797361 | rural | forest | *U. glabra* | 18.07.2016 | no | **8201** |
| 70 | Estonia | Heimtali | 58.332433,25.515583 | rural | forest | *U. glabra* | 20.07.2016 | no | **8304** |
| 71 | Estonia | Heimtali | 58.333100,25.523533 | rural | forest | *U. glabra* | 20.07.2016 | no | **8312** |
| 72 | Estonia | Heimtali | 58.332950,25.526100 | rural | forest | *U. glabra* | 20.07.2016 | no | **8315** |
| 73 | Estonia | Heimtali | 58.332450,25.524683 | rural | forest | *U. glabra* | 20.07.2016 | no | **8316** |
| 74 | Estonia | Heimtali | 58.323350,25.537133 | rural | forest | *U. glabra* | 20.07.2016 | no | **8317** |
| 75 | Russia | St. Petersburg | 59.987599,30.339253 | urban space | park | *U. glabra* | 10.06.2016 | yes | **6011** |
| 76 | Russia | St. Petersburg | 59.987667,30.341405 | urban space | park | *U. glabra* | 10.06.2016 | yes | **6014** |
| 77 | Russia | St. Petersburg | 60.012921,30.369612 | urban space | street | *U. glabra* | 11.06.2016 | yes | **6024** |
| 78 | Russia | Vyborg | 60.707867,28.750583 | urban space | street | *U. glabra* | 23.07.2016 | yes | **6755** |
| 79 | Russia | Tsarskoje Selo | 59.723183,30.393283 | urban space | park | *U. glabra* | 22.07.2016 | yes | **6745** |

| | Country | Name of the site | Coordinates | Environment | Habitat | Host | Sampling date | Canopy dieback | ID No |
|---|---|---|---|---|---|---|---|---|---|
| 80 | Russia | Tsarskoje Selo | 59.720267,30.398417 | urban space | street | *U. glabra* | 22.07.2016 | yes | **6752** |
| 81 | Russia | St. Petersburg | 60.015767,30.371183 | urban space | street | *U. glabra* | 21.07.2016 | yes | **6743** |
| 82 | Russia | St. Petersburg | 60.015167,30.371000 | urban space | street | *U. glabra* | 21.07.2016 | yes | **6736** |
| 83 | Russia | St. Petersburg | 59.945867,30.377317 | urban space | park | *U. glabra* | 21.07.2016 | yes | **6719** |
| 84 | Russia | St. Petersburg | 59.945067,30.375517 | urban space | park | *U. glabra* | 21.07.2016 | yes | **6716** |
| 85 | Russia | St. Petersburg | 59.987523,30.338337 | urban space | park | *U. glabra* | 14.06.2016 | yes | **6013** |
| 86 | Russia | Vyborg | 60.707867,28.750583 | urban space | street | *U. glabra* | 23.07.2016 | yes | **6759** |
| 87 | Russia | Vyborg | 60.706950,28.750250 | urban space | street | *U. glabra* | 23.07.2016 | yes | **6754** |
| 88 | Russia | Vyborg | 60.706950,28.750250 | urban space | street | *U. glabra* | 23.07.2016 | yes | **6758** |
| 89 | Russia | St. Petersburg | 59.920283,30.350833 | urban space | street | *U. glabra* | 21.07.2016 | yes | **6705** |
| 90 | Russia | Vyborg | 60.703650,28.780133 | urban space | street | *U. glabra* | 23.07.2016 | yes | **6765** |
| 91 | Russia | Vyborg | 60.703650,28.780133 | urban space | street | *U. glabra* | 23.07.2016 | yes | **6766** |
| 92 | Russia | Vyborg | 60.703650,28.780133 | urban space | street | *U. glabra* | 23.07.2016 | yes | **6767** |
| 93 | Russia | Primorskaja | 60.697100,28.776800 | urban space | street | *U. glabra* | 23.07.2016 | yes | **6769** |

| | Country | Name of the site | Coordinates | Environment | Habitat | Host | Sampling date | Canopy dieback | ID No |
|---|---|---|---|---|---|---|---|---|---|
| 94 | Russia | Vyborg | 60.703650,28.780133 | urban space | street | *U. glabra* | 23.07.2016 | no | **8244** |
| 95 | Russia | St. Petersburg | 60.015767,30.371183 | urban space | street | *U. glabra* | 21.07.2016 | no | **8232** |
| 96 | Russia | St. Petersburg | 60.015767,30.371183 | urban space | street | *U. glabra* | 21.07.2016 | no | **8235** |
| 97 | Russia | St. Petersburg | 60.015167,30.371000 | urban space | street | *U. glabra* | 21.07.2016 | no | **8234** |
| 98 | Russia | St. Petersburg | 59.945067,30.375517 | urban space | park | *U. glabra* | 21.07.2016 | no | **8230** |
| 99 | Russia | St. Petersburg | 59.938083,30.368383 | urban space | park | *U. glabra* | 21.07.2016 | no | **8228** |
| 100 | Estonia | Surju | 58.238303,24.708607 | rural | street | *U. laevis* | 24.08.2015 | yes | **4592** |
| 101 | Estonia | Kurgja | 58.663388,25.256415 | rural | park | *U. laevis* | 19.07.2015 | yes | **4360** |
| 102 | Estonia | Tihemetsa | 58.149517,25.047600 | rural | park | *U. laevis* | 10.07.2016 | yes | **6399** |
| 103 | Estonia | Surju | 58.238303,24.708607 | rural | park | *U. laevis* | 24.08.2015 | yes | **4591** |
| 104 | Estonia | Viljandi | 58.360388,25.592121 | urban space | park | *U. laevis* | 01.07.2015 | yes | **4296** |
| 105 | Estonia | Kurgja | 58.663192, 25.255501 | rural | park | *U. laevis* | 19.07.2015 | yes | **4359** |
| 106 | Estonia | Kurgja | 58.663388,25.256415 | rural | park | *U. laevis* | 19.07.2015 | yes | **4361** |
| 107 | Estonia | Surju | 58.238303,24.708607 | rural | park | *U. laevis* | 24.08.2015 | yes | **4597** |
| 108 | Estonia | Tartu | 58.389046,26.697989 | urban space | park | *U. laevis* | 17.06.2016 | yes | **8290** |
| 109 | Estonia | Tihemetsa | 58.148350,25.046417 | rural | park | *U. laevis* | 10.07.2016 | no | **8160** |
| 110 | Estonia | Tihemetsa | 58.148317,25.046250 | rural | park | *U. laevis* | 10.07.2016 | no | **8161** |
| 111 | Estonia | Tihemetsa | 58.148450,25.046467 | rural | park | *U. laevis* | 10.07.2016 | no | **8162** |
| 112 | Estonia | Tihemetsa | 58.148383,25.046583 | rural | park | *U. laevis* | 10.07.2016 | no | **8163** |
| 113 | Estonia | Tihemetsa | 58.148383,25.046583 | rural | park | *U. laevis* | 10.07.2016 | no | **8164** |

| | Country | Name of the site | Coordinates | Environment | Habitat | Host | Sampling date | Canopy dieback | ID No |
|---|---|---|---|---|---|---|---|---|---|
| 114 | Estonia | Tartu | 58.388883,26.724617 | urban space | park | *U. laevis* | 03.07.2016 | no | **8288** |
| 115 | Estonia | Tartu | 58.389141, 26.698077 | urban space | park | *U. laevis* | 17.06.2016 | no | **8291** |
| 116 | Estonia | Tartu | 58.386767,26.695867 | urban space | park | *U. laevis* | 17.06.2016 | no | **8293** |
| 117 | Estonia | Tartu | 58.386867,26.695867 | urban space | park | *U. laevis* | 17.06.2016 | no | **8294** |
| 118 | Estonia | Tartu | 58.387733,26.697417 | urban space | park | *U. laevis* | 17.06.2016 | no | **8295** |
| 119 | Estonia | Heimtali | 58.332283,25.522967 | rural | forest | *U. laevis* | 20.07.2016 | yes | **6559** |
| 120 | Estonia | Heimtali | 58.333250,25.523867 | rural | forest | *U. laevis* | 20.07.2016 | yes | **6561** |
| 121 | Estonia | Poruni | 59.140733,27.816617 | rural | forest | *U. laevis* | 18.07.2016 | no | **8198** |
| 122 | Estonia | Poruni | 59.175400,27.796900 | rural | forest | *U. laevis* | 18.07.2016 | no | **8199** |
| 123 | Estonia | Poruni | 59.175800,27.796650 | rural | forest | *U. laevis* | 18.07.2016 | no | **8200** |
| 124 | Estonia | Luua | 58.648560,26.599916 | rural | forest | *U. laevis* | 31.07.2016 | no | **8246** |
| 125 | Estonia | Luua | 58.648560,26.599916 | rural | forest | *U. laevis* | 31.07.2016 | no | **8248** |
| 126 | Estonia | Luua | 58.648560,26.599916 | rural | forest | *U. laevis* | 31.07.2016 | no | **8250** |
| 127 | Estonia | Luua | 58.648560,26.599916 | rural | forest | *U. laevis* | 31.07.2016 | no | **8251** |
| 128 | Estonia | Luua | 58.648560,26.599916 | rural | forest | *U. laevis* | 31.07.2016 | no | **8252** |
| 129 | Estonia | Heimtali | 58.332100,25.522183 | rural | forest | *U. laevis* | 20.07.2016 | no | **8308** |
| 130 | Estonia | Heimtali | 58.332283,25.522967 | rural | forest | *U. laevis* | 20.07.2016 | no | **8309** |
| 131 | Russia | St. Petersburg | 59.989471,30.341650 | urban space | park | *U. laevis* | 10.06.2016 | yes | **6008** |
| 132 | Russia | St. Petersburg | 59.987744,30.339854 | urban space | park | *U. laevis* | 10.06.2016 | yes | **6009** |
| 133 | Russia | St. Petersburg | 59.987453,30.338377 | urban space | park | *U. laevis* | 10.06.2016 | yes | **6012** |

| | Country | Name of the site | Coordinates | Environment | Habitat | Host | Sampling date | Canopy dieback | ID No |
|---|---|---|---|---|---|---|---|---|---|
| 134 | Russia | St. Petersburg | 59.945350,30.374617 | urban space | park | *U. laevis* | 21.07.2016 | yes | **6715** |
| 135 | Russia | St. Petersburg | 60.028669,30.387808 | urban space | park | *U. laevis* | 11.06.2016 | yes | **6023** |
| 136 | Russia | St. Petersburg | 60.028267,30.387581 | urban space | park | *U. laevis* | 11.06.2016 | yes | **6020** |
| 137 | Russia | St. Petersburg | 60.010017,30.345800 | urban space | street | *U. laevis* | 21.07.2016 | yes | **6722** |
| 138 | Russia | Vyborg | 60.707867,28.750583 | urban space | street | *U. laevis* | 23.07.2016 | yes | **6760** |
| 139 | Russia | St. Petersburg | 59.938183,30.367750 | urban space | park | *U. laevis* | 21.07.2016 | yes | **6708** |
| 140 | Russia | St. Petersburg | 59.938183,30.367750 | urban space | park | *U. laevis* | 21.07.2016 | yes | **6707** |
| 141 | Russia | St. Petersburg | 60.028519,30.387876 | urban space | park | *U. laevis* | 11.06.2016 | yes | **6021** |
| 142 | Russia | St. Petersburg | 60.028632,30.388020 | urban space | park | *U. laevis* | 11.06.2016 | yes | **6022** |
| 143 | Russia | St. Petersburg | 60.028361,30.387510 | urban space | park | *U. laevis* | 11.06.2016 | yes | **6019** |
| 144 | Russia | St. Petersburg | 59.987523,30.338337 | urban space | park | *U. laevis* | 10.06.2016 | yes | **6010** |
| 145 | Russia | Vyborg | 60.706950,28.750250 | urban space | street | *U. laevis* | 23.07.2016 | no | **8240** |
| 146 | Russia | Vyborg | 60.733767,28.726833 | urban space | park | *U. laevis* | 23.07.2016 | no | **8239** |
| 147 | Russia | Tsarskoje Selo | 59.720350,30.399117 | urban space | street | *U. laevis* | 22.07.2016 | no | **8238** |

| | Country | Name of the site | Coordinates | Environment | Habitat | Host | Sampling date | Canopy dieback | ID No |
|---|---|---|---|---|---|---|---|---|---|
| 148 | Russia | Tsarskoje Selo | 59.724950,30.404917 | urban space | street | *U. laevis* | 22.07.2016 | no | **8236** |
| 149 | Estonia | Tartu | 58.360200,26.687383 | urban space | street | hybrid | 13.07.2016 | yes | **6371** |
| 150 | Estonia | Tartu | 58.360450,26.688033 | urban space | street | hybrid | 13.07.2016 | yes | **6405** |
| 151 | Estonia | Tartu | 58.360167,26.687583 | urban space | street | hybrid | 13.07.2016 | yes | **6370** |
| 152 | Estonia | Tartu | 58.360283,26.687867 | urban space | street | hybrid | 13.07.2016 | yes | **6403** |
| 153 | Estonia | Tartu | 58.360433,26.687967 | urban space | street | hybrid | 13.07.2016 | yes | **6404** |
| 154 | Estonia | Tartu | 58.360200,26.687783 | urban space | street | hybrid | 13.07.2016 | no | **8287** |
| 155 | Estonia | Tartu | 58.360217,26.687650 | urban space | street | hybrid | 13.07.2016 | no | **8298** |
| 156 | Estonia | Tartu | 58.360150,26.687433 | urban space | street | hybrid | 13.07.2016 | no | **8299** |
| 157 | Estonia | Tartu | 58.360100,26.687283 | urban space | street | hybrid | 13.07.2016 | no | **8346** |
| 158 | Russia | St. Petersburg | 59.921782,30.359725 | urban space | street | hybrid | 11.06.2016 | yes | **6026** |
| 159 | Russia | St. Petersburg | 59.919892,30.358386 | urban space | street | hybrid | 11.06.2016 | yes | **6032** |
| 160 | Russia | St. Petersburg | 59.919583,30.358391 | urban space | street | hybrid | 11.06.2016 | yes | **6034** |
| 161 | Russia | St. Petersburg | 59.918321,30.357535 | urban space | street | hybrid | 11.06.2016 | yes | **6039** |

| | Country | Name of the site | Coordinates | Environment | Habitat | Host | Sampling date | Canopy dieback | ID No |
|---|---|---|---|---|---|---|---|---|---|
| 162 | Russia | St. Petersburg | 59.917645,30.357109 | urban space | street | hybrid | 11.06.2016 | yes | **6043** |
| 163 | Russia | St. Petersburg | 59.916172,30.356261 | urban space | street | hybrid | 11.06.2016 | yes | **6048** |
| 164 | Russia | St. Petersburg | 59.945383,30.376967 | urban space | park | hybrid | 21.07.2016 | yes | **6717** |
| 165 | Russia | St. Petersburg | 59.938067,30.369083 | urban space | park | hybrid | 21.07.2016 | yes | **6710** |
| 166 | Russia | St. Petersburg | 59.921983,30.359933 | urban space | street | hybrid | 11.06.2016 | yes | **6025** |
| 167 | Russia | St. Petersburg | 59.921242,30.359349 | urban space | street | hybrid | 11.06.2016 | yes | **6028** |
| 168 | Russia | St. Petersburg | 59.921147,30.359269 | urban space | street | hybrid | 11.06.2016 | yes | **6029** |
| 169 | Russia | St. Petersburg | 59.920020,30.358658 | urban space | street | hybrid | 11.06.2016 | yes | **6030** |
| 170 | Russia | St. Petersburg | 59.919990,30.358486 | urban space | street | hybrid | 11.06.2016 | yes | **6031** |
| 171 | Russia | St. Petersburg | 59.919843,30.358361 | urban space | street | hybrid | 11.06.2016 | yes | **6033** |
| 172 | Russia | St. Petersburg | 59.918390,30.357601 | urban space | street | hybrid | 11.06.2016 | yes | **6038** |
| 173 | Russia | St. Petersburg | 59.918231,30.357405 | urban space | street | hybrid | 11.06.2016 | yes | **6040** |
| 174 | Russia | St. Petersburg | 59.918068,30.357419 | urban space | street | hybrid | 11.06.2016 | yes | **6041** |
| 175 | Russia | St. Petersburg | 59.915688, 30.355954 | urban space | street | hybrid | 11.06.2016 | yes | **6044** |

| | Country | Name of the site | Coordinates | Environment | Habitat | Host | Sampling date | Canopy dieback | ID No |
|---|---|---|---|---|---|---|---|---|---|
| 176 | Russia | St. Petersburg | 59.915461,30.355694 | urban space | street | hybrid | 11.06.2016 | yes | **6045** |
| 177 | Russia | St. Petersburg | 59.945867,30.377317 | urban space | park | hybrid | 21.07.2016 | no | **8233** |
| 178 | Russia | St. Petersburg | 59.919829,30.358487 | urban space | street | hybrid | 11.06.2016 | no | **8221** |
| 179 | Russia | St. Petersburg | 59.919619,30.358417 | urban space | street | hybrid | 11.06.2016 | no | **8223** |
| 180 | Russia | St. Petersburg | 59.918009,30.357371 | urban space | street | hybrid | 11.06.2016 | no | **8225** |
| 181 | Russia | St. Petersburg | 59.917825,30.357296 | urban space | street | hybrid | 11.06.2016 | no | **8226** |
| 182 | Russia | St. Petersburg | 59.920283,30.350833 | urban space | park | hybrid | 21.07.2016 | no | **8227** |
| 183 | Russia | St. Petersburg | 59.936733,30.365883 | urban space | park | hybrid | 21.07.2016 | no | **8229** |

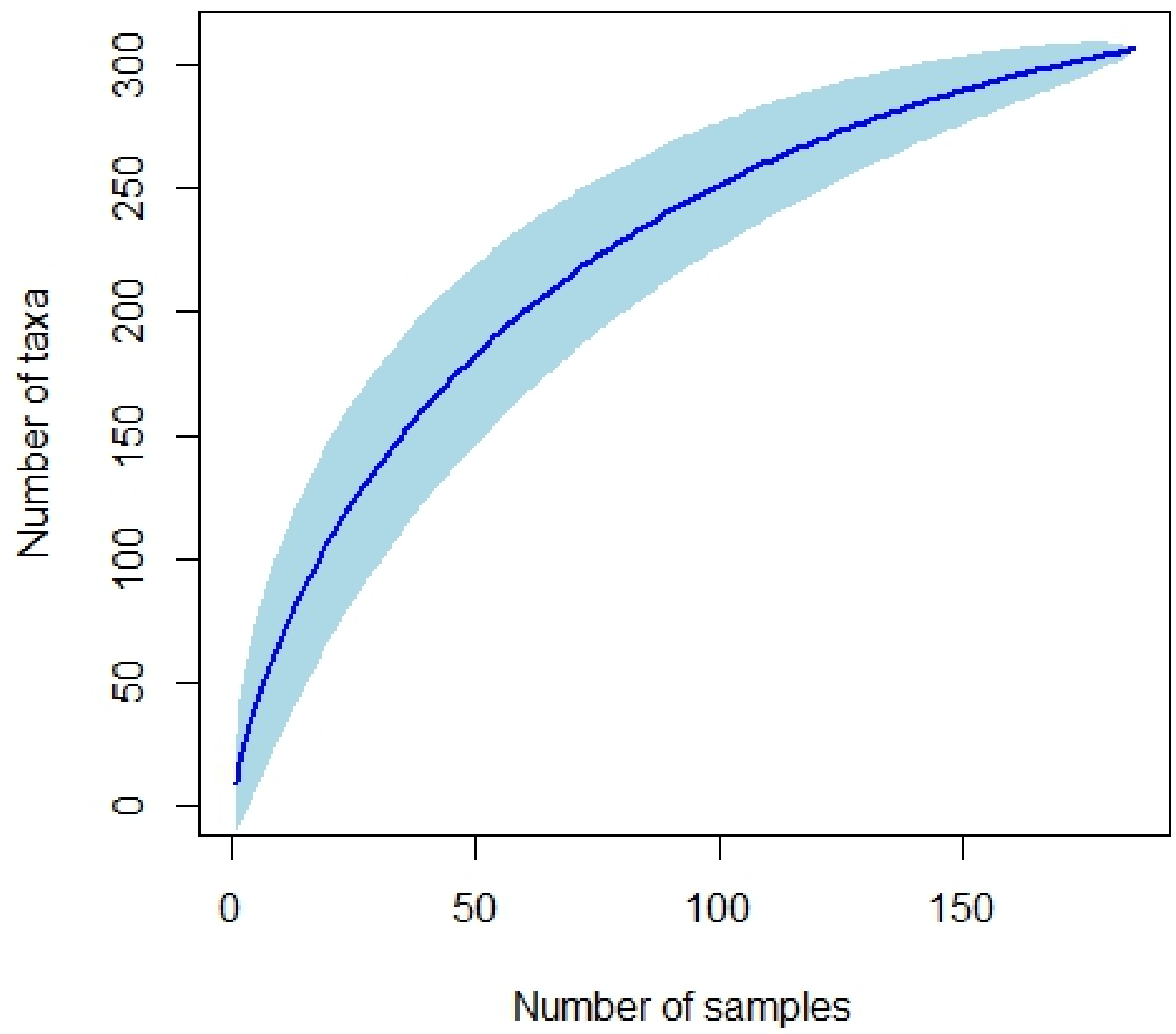


a

Fig. S1. Species accumulation curve across the entire dataset.

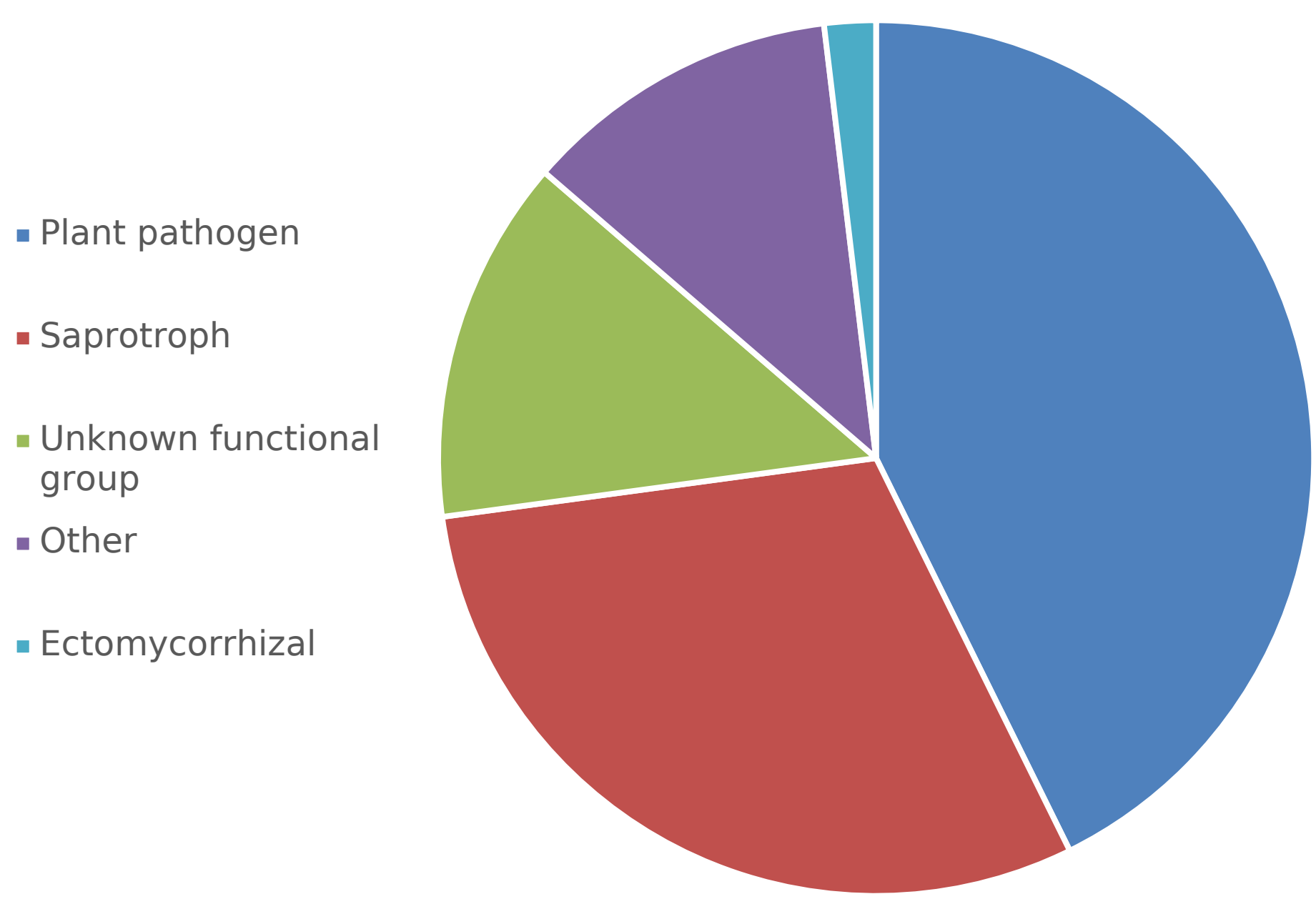


Fig. S2. The proportions of functional groups of fungi across three different species of *Ulmus*

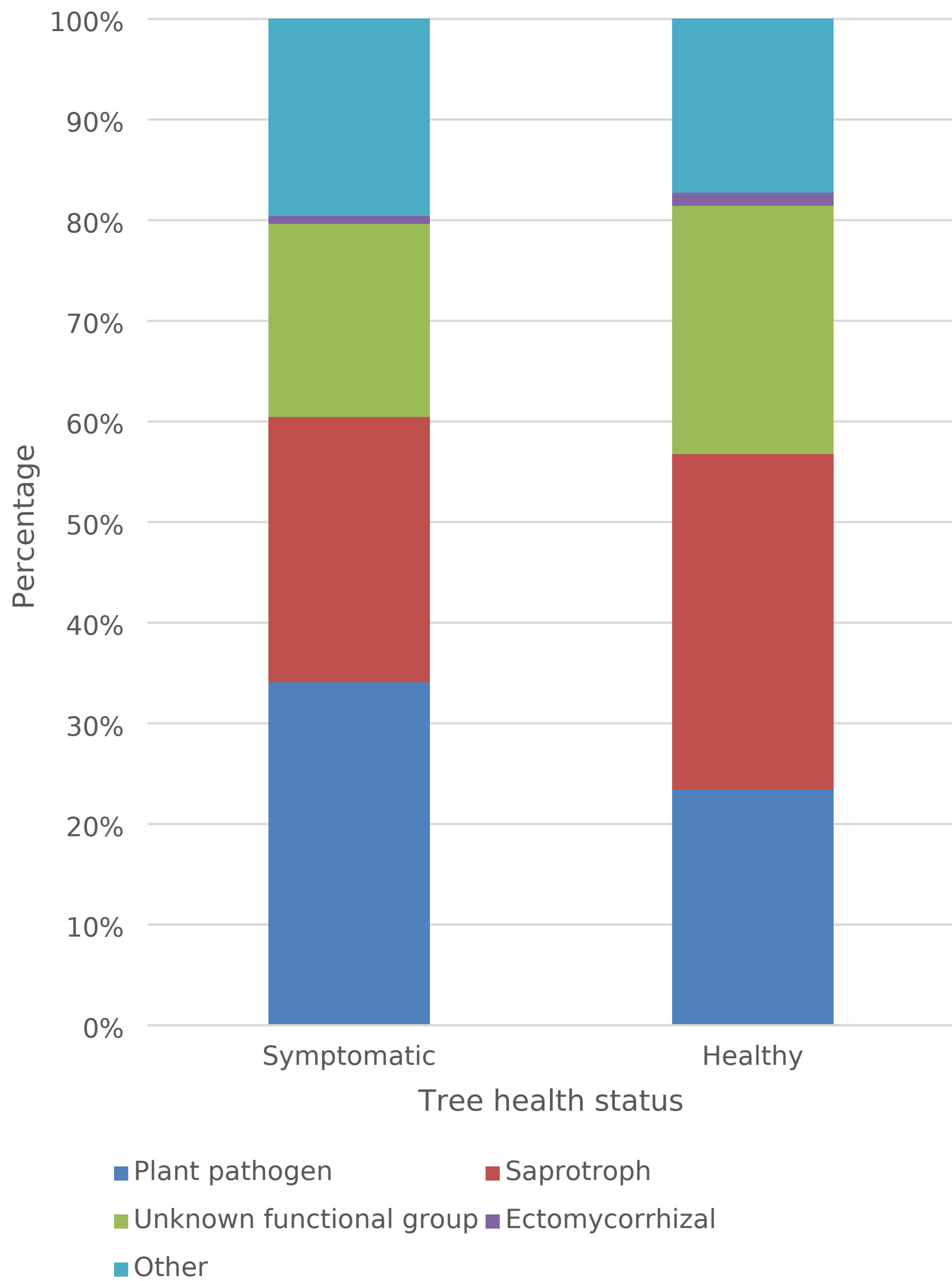
100%
90%
80%
70%
60%
50%
40%
30%
20%
10%
0%
Percentage
Symptomatic
Healthy
Tree health status
Plant pathogen
Saprotroph
Unknown functional group
Ectomycorrhizal
Other

a

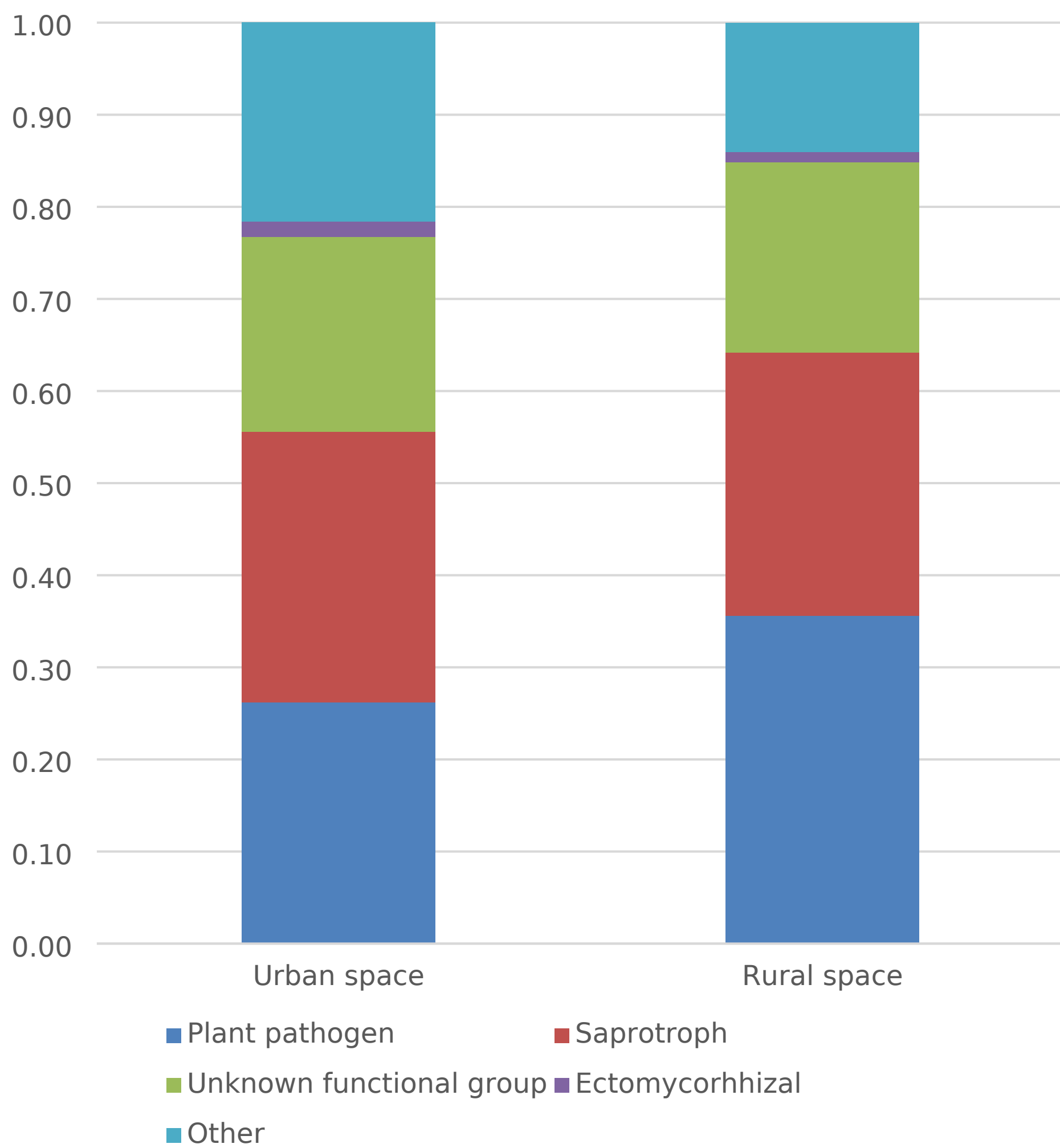


b

Fig. S3. The proportions of functional groups of fungi on visually healthy and symptomatic trees of *Ulmus* (a) and between urban and rural spaces (b).

**Author Contribution**

L.J., R.D. and A.A. wrote the main manuscript text and A.A. prepared figures 2-6, L.J. prepared figure 1. L.J. and A.S collected the samples. All authors reviewed the manuscript.

**~~Acknowledgments~~Funding**

This study was supported by the Estonian Research Council grant PRG1615~~,~~ and co-funded by the European Union and Ministry of Education and Research via project TEMTA22 (High-quality wood from managed forests and plantations) and supported by COST Action Urban Tree Guard (CA20132).

**Data ~~a~~Availability**

The demultiplexed raw sequencing data is available in SRA (Sequence read archive) under accession number PRJNA1209654.

## Declarations

## Conflict of Interest

The authors declare no competing interests.

## References


1. Kļaviņa, D., et al., *Host-associated intraspecific phenotypic variation in the saprobic fungus Phlebiopsis gigantea.* Microbial Ecology, 2023. **1**: p. 1-9.
2. Agan, A., et al., *Seasonal dynamics of fungi associated with healthy and diseased pinus sylvestris needles in Northern Europe.* Microorganisms, 2021. **9**(8): p. 1757-1757.
3. Kovalchuk, A., et al., *Mycobiome analysis of asymptomatic and symptomatic Norway spruce trees naturally infected by the conifer pathogens Heterobasidion spp.* Environmental Microbiology Reports, 2018. **10**(5): p. 532-541.
4. Nguyen, N.H., et al., *FUNGuild: an open annotation tool for parsing fungal community datasets by ecological guild.* Fungal Ecology, 2016. **20**: p. 241-248.
5. Agan, A., et al., *The relationship between fungal diversity and invasibility of a foliar niche—the case of ash dieback.* Journal of Fungi, 2020. **6**(3): p. 1-27.
6. Schlegel, M., V. Queloz, and T.N. Sieber, *The endophytic mycobiome of European ash and Sycamore maple leaves - geographic patterns, host specificity and influence of ash dieback.* Frontiers in Microbiology, 2018. **9**(OCT).
7. Cross, H., et al., *Fungal diversity and seasonal succession in ash leaves infected by the invasive ascomycete Hymenoscyphus fraxineus.* New Phytologist, 2017. **213**(3): p. 1405-1417.
8. Marčiulynas, A., et al., *Fungal communities in leaves and roots of healthy-looking and diseased Ulmus glabra.* Microorganisms, 2022. **10**(11): p. 2228-2228.
9. Martín, J.A., et al., *Resistance to Dutch elm disease reduces presence of xylem endophytic fungi in elms (Ulmus spp.).* PLoS ONE, 2013. **8**(2): p. e56987-e56987.
10. Kowalski, T., *Endophytic fungi: VI. Mycobiota in living symptomless leaves of Ulmus glabra and in necrotic tissues associated with gall-making insects.* Phytopathologia Polonica, 2004. **32**: p. 61-73.
11. Terhonen, E., F. Oskay, and R. Kasanen, *Editorial: the effect of mycobiomes on health of forest trees.* Frontiers in Microbiology, 2023. **14**: p. 544-544.
12. Martínez-Arias, C., et al., *Enhancement of Populus alba tolerance to Venturia tremulae upon inoculation with endophytes showing in vitro biocontrol potential.* European Journal of Plant Pathology, 2019. **153**(4): p. 1031-1042.
13. Martín, J.A., et al., *Strong in vitro antagonism by elm xylem endophytes is not accompanied by temporally stable in planta protection against a vascular pathogen under field conditions.* European Journal of Plant Pathology, 2015. **142**(1): p. 185-196.
14. Witzell, J. and J.A. Martín, *Endophytes and forest health*, in *endophytes of forest trees: biology and applications*, A.M. Pirttilä and A.C. Frank, Editors. 2018, Springer International Publishing: Cham. p. 261-282.

15. Caudullo, G. and D. De Rigo, *Ulmus - elms in Europe: distribution, habitat, usage and threats*, in *European Atlas of Forest Tree Species.*, J. San-Miguel-Ayanz, et al., Editors. 2016: Luxembourg.
16. Kalamees, K., *Roosa võrkheinik: seenharuldus jalakal (wrinkled peach: rare fungus on elms) [In Estonian].* Eesti Loodus (Estonian Nature), 2011. **62**(10): p. 41-41.
17. Thor, G., P. Johansson, and M.T. Jönsson, *Lichen diversity and red-listed lichen species relationships with tree species and diameter in wooded meadows.* Biodiversity and Conservation, 2010. **19**: p. 2307-2328.
18. Jüriado, I., J. Liira, and J. Paal, *Diversity of epiphytic lichens in boreo-nemoral forests on the North-Estonian limestone escarpment: the effect of tree level factors and local environmental conditions.* Lichenologist, 2009. **41**(1): p. 81-96.
19. Corfixen, P. and E. Parmasto, *<i>Hymenochaete ulmicola</i> sp. nov. (Hymenochaetales).* Mycotaxon, 2005. **91**: p. 465–469-465–469.
20. Hiemstra, J.A. and J. Buiteveld. *New perspectives for the use of elms as street trees*. 2010. International Society for Horticultural Science (ISHS), Leuven, Belgium.
21. Ignatieva, M. and G. Konechnaya, *Floristic investigations of historical parks in floristic investigations of historical parks in St. Petersburg, Russia.* Urban Habitats, 2004. **2**(1): p. 174-216.
22. Ignatieva, M., G. Konechnaya, and G. Stewart, *St. Petersburg*, in *Plants and habitats of European cities*. 2011, Springer New York: New York, NY. p. 407-452.
23. Mart□n, J.A., et al., *Ecological factors in Dutch elm disease complex in Europe-a review.* Ecological Bulletins, 2010(53): p. 209-224.
24. Bernier, L., *Chapter 16 - Dutch elm disease*, in *Forest microbiology*, F.O. Asiegbu and A. Kovalchuk, Editors. 2022, Academic Press. p. 291-309.
25. Jürisoo, L., et al., *The extensive damage to elms by Dutch elm disease agents and their hybrids in northwestern Russia.* Urban Forestry & Urban Greening, 2021. **63**: p. 127214-127214.
26. Martín, J.A., et al., *Complexities underlying the breeding and deployment of Dutch elm disease resistant elms.* New Forests, 2021. **7**: p. 1-36.
27. Santini, A., et al., *Breeding against Dutch elm disease adapted to the Mediterranean climate.* Euphytica, 2008. **163**(1): p. 45-56.
28. Jürisoo, L., et al., *Health of elms and Dutch elm disease in Estonia.* European Journal of Plant Pathology, 2019. **154**(3): p. 823-841.
29. Jürisoo, L., A. Padari, and R. Drenkhan, *Spread and riskiness of Dutch elm disease in Estonia (Jalakasurma levikust ja ohtlikkusest Eestis) [In Estonian].* Forestry Studies | Metsanduslikud Uurimused, 2021. **74**: p. 88–111.
30. Smith, S.M. and J. Hulcr, *<i>Scolytus</i> and other economically important bark and ambrosia beetles*, in *Bark beetles biology and ecology of native and invasive species*, F.E. Vega and R.W. Hofstetter, Editors. 2015, Academic Press. p. 495-531.
31. Pines, I.L. and R.P. Westwood, *A mark-recapture technique for the Dutch elm disease vector the native elm bark beetle, <i>Hylurgopinus rufipes</i> (Coleoptera: Scolytidae).* Arboriculture & Urban Forestry, 2008. **34**(2): p. 116-122.
32. Jürisoo, L., et al., *Vectors of Dutch elm disease in Northern Europe.* Insects, 2021. **12**: p. 393-393.
33. Campanella, T.J., *Republic of shade: New England and the American elm | Environment & Society Portal*. 2011: Yale University Press. 240-240.

34. Ouellette, G.B., et al., *Fine structure of the extracellular sheath and cell walls in Ophiostoma novo-ulmi growing on various substrates.* Canadian Journal of Microbiology, 1999. **45**(7): p. 582-597.
35. Pihlgren, A.H., Tomas; Aronsson, Mora; Dahlberg, Anders; Edqvist, M; Johansson, G; Krikorev, Michael; Thor, Göran, *Var femte växt och svamp är med på nya rödlistan.* Svensk Botanisk Tidskrift, 2010. **104**(4): p. 210-226.
36. Drenkhan, R., et al., *The spread and danger of Dutch elm disease in Estonian forests and green areas [In Estonian]*. 2020, Estonian University of Life Sciences. p. 40.
37. Buisman, C., *Three species of Botydiplodia (Sacc.) on elm trees in the United States.* Journal of the Arnold Arboretum, 1931. **12**: p. 289-296.
38. Bartnik, C., et al., *The first record of Botryodiplodia canker in Poland.* Forest Pathology, 2019. **49**(4): p. e12528-e12528.
39. Bartnik, C., J. Michalcewicz, and M. Ciach, *Infection potential of Botryodiplodia hypodermia, the causal agent of elm canker.* Journal of Plant Pathology, 2022. **104**(3): p. 1123-1128.
40. Addison, S.L., et al., *Partner or perish: tree microbiomes and climate change.* Trends in Plant Science, 2024. **29**(9): p. 1029-1040.
41. Berg, G., et al., *Plant microbial diversity is suggested as the key to future biocontrol and health trends.* FEMS Microbiology Ecology, 2017. **93**(5).
42. Sahai, P. and V. Kumar, *Microbiome: effect on plant system, current application and future aspect*, in *In vitro plant breeding towards novel agronomic traits: biotic and abiotic stress tolerance*. 2019. p. 119-134.
43. Spooren, J., et al., *Plant-driven assembly of disease-suppressive soil microbiomes.* Annual Review of Phytopathology, 2024. **62**(1): p. 1-30.
44. Agler, M.T., et al., *Microbial hub taxa link host and abiotic factors to plant microbiome variation.* PLOS Biology, 2016. **14**(1): p. e1002352-e1002352.
45. Vandenkoornhuyse, P., et al., *The importance of the microbiome of the plant holobiont.* New Phytologist, 2015. **206**(4): p. 1196-1206.
46. Büchel, K., et al., *Elm defence against herbivores and pathogens: morphological, chemical and molecular regulation aspects*, in *Phytochemistry Reviews*. 2016.
47. Kumar, V., et al., *Endophytes as potential plant growth promoters in forestry: recent advances and perspectives*, in *Microbes in Agri-Forestry Biotechnology*. 2022. p. 241-262.
48. Dar, Z.A., et al., *Potential role of endophytes for sustainable environment*, in *Research anthology on emerging techniques in environmental remediation*. 2022. p. 177-194.
49. Durodola, B., et al., *Beyond the surface: exploring the mycobiome of Norway spruce under drought stress and with Heterobasidion parviporum.* BMC Microbiology, 2023. **23**(1).
50. Martínez□arias, C., et al., *Priming of plant defenses against ophiostoma novo□ulmi by elm (Ulmus minor mill.) fungal endophytes.* Journal of Fungi, 2021. **7**(9): p. 687-687.
51. Wen, Z., E. Terhonen, and F.O. Asiegbu, *The dark septate endophyte Phialocephala sphaeroides confers growth fitness benefits and mitigates pathogenic effects of Heterobasidion on Norway spruce.* Tree Physiology, 2022. **42**(4): p. 891-906.
52. Blumenstein, K., et al., *Nutritional niche overlap potentiates the use of endophytes in biocontrol of a tree disease.* BioControl, 2015. **60**(5): p. 655-667.
53. Nogales, A., et al., *Can functional hologenomics aid tackling current challenges in plant breeding?* Briefings in Functional Genomics, 2016. **15**(4): p. 288-297.

54. Blumenstein, K., et al., *Methods for studying the forest tree microbiome*, in *Forest microbiology*, F.O. Asiegbu and A. Kovalchuk, Editors. 2021, Academic Press. p. 35-58.
55. Macaya-Sanz, D., et al., *Core endophytic mycobiome in Ulmus minor and its relation to Dutch elm disease resistance.* Frontiers in Plant Science, 2023. **14**.
56. Tedersoo, L., et al., *Global diversity and geography of soil fungi.* Science, 2014. **346**(6213).
57. Tedersoo, L. and S. Anslan, *Towards PacBio□based pan□eukaryote metabarcoding using full□length ITS sequences.* Environmental Microbiology Reports, 2019. **11**(5): p. 659-668.
58. Tedersoo, L., A. Tooming-Klunderud, and S. Anslan, *PacBio metabarcoding of fungi and other eukaryotes: errors, biases and perspectives.* New Phytologist, 2018. **217**(3): p. 1370-1385.
59. Anslan, S., et al., *PipeCraft: flexible open-source toolkit for bioinformatics analysis of custom high-throughput amplicon sequencing data.* Molecular Ecology Resources, 2017. **17**(6): p. e234-e240.
60. Schloss, P.D., et al., *Introducing mothur: open-source, platform-independent, community-supported software for describing and comparing microbial communities.* Applied and Environmental Microbiology, 2009. **75**(23): p. 7537-7541.
61. Edgar, R.C., et al., *UCHIME improves sensitivity and speed of chimera detection.* Bioinformatics, 2011. **27**(16): p. 2194-2200.
62. Fu, L., et al., *CD-HIT: accelerated for clustering the next-generation sequencing data.* Bioinformatics, 2012. **28**(23): p. 3150-3152.
63. Kõljalg, U., et al., *Towards a unified paradigm for sequence-based identification of fungi.* Molecular Ecology, 2013. **22**(21): p. 5271-5277.
64. Hammer, Ø., D.A.T. Harper, and P.D. Ryan, *Past: paleontological statistics software package for education and data analysis.* Palaeontologia Electronica, 2001. **4**(1): p. 178-178.
65. Bates, D., et al., *Fitting linear mixed-effects models using lme4.* Journal of Statistical Software, 2015. **67**(1): p. 1-48.
66. Põlme, S., et al., *FungalTraits: a user-friendly traits database of fungi and fungus-like stramenopiles.* Fungal Diversity, 2020. **105**(1): p. 1-16.
67. Anderson, M.J., R.N. Gorley, and K.R. Clarke, *PERMANOVA+ for PRIMER: guide to software and statistical methods*. 2008: Plymouth, Devon. p. 214-214.
68. Clarke, K., *PRIMER V6: user manual.* Tutorial/Primer-E Ltd, 2006. **190**.
69. Griffith, D.M., J.A. Veech, and C.J. Marsh, *cooccur: probabilistic species co-occurrence analysis in R.* Journal of Statistical Software, Code Snippets, 2016. **69**(2): p. 1 - 17.
70. Lamit, L.J., et al., *Tree genotype and genetically based growth traits structure twig endophyte communities.* American Journal of Botany, 2014. **101**(3): p. 467-478.
71. Juutilainen, K., et al., *Resource use of wood-inhabiting fungi in different boreal forest types.* Fungal Ecology, 2017. **27**: p. 96-106.
72. Vemić, A., *The most important fungi on wych elm (Ulmus glabra) trees in Montenegro.* Agriculture & Forestry, 2022. **68**(3): p. 71-82.
73. Rähn, E., et al., *Rapid shift of soil fungal community compositions after clear-cutting in hemiboreal coniferous forests.* Forest Ecology and Management, 2023. **544**: p. 121211.

74. Lourenco, A. and L.J. Hutchison, *A survey of branch- and twig-inhabiting fungi isolated from declining urban elms in Thunder Bay, Ontario.* Canadian Journal of Plant Pathology, 2008. **30**(2): p. 335-338.
75. Romeralo, C., et al., *Pine species determine fungal microbiome composition in a common garden experiment.* Fungal Ecology, 2022. **56**: p. 101137.
76. Crous, P.W. and J.Z. Groenewald, *The genera of fungi - G 4: Camarosporium and Dothiora.* IMA Fungus, 2017. **8**(1): p. 131-152.
77. Menkis, A., et al., *Archaeorhizomyces borealis sp. nov. and a sequence-based classification of related soil fungal species.* Fungal Biology, 2014. **118**(12): p. 943-955.
78. Turaliyeva, M., et al., *Base of biofungicide creation for protection of plants Ulmus pumilla L. against fungus diseases in conditions of urbanodendroflora in Southern Kazakhstan.* Journal of Biotechnology, 2014. **185**: p. S115-S115.
79. de Gruyter, H., et al., *Redisposition of phoma-like anamorphs in pleosporales.* Studies in Mycology, 2013. **75**: p. 1-36.
80. Zhang, X., et al., *Morphological, physiological, and transcriptional responses to drought stress in sensitive and tolerant elm (Ulmus pumila L.) varieties.* Forests, 2025. **16**(1).
81. Santini, A., et al., *Genotype × environment interaction and growth stability of several elm clones resistant to Dutch elm disease.* Forest Ecology and Management, 2010. **260**(6): p. 1017-1025.
82. Martín-García, J., et al., *Factors influencing endophytic communities in poplar plantations.* Silva Fennica, 2011. **45**(2): p. 169-180.
83. Hofmann, B., et al., *Habitat and tree species identity shape aboveground and belowground fungal communities in central European forests.* Frontiers in Microbiology, 2023. **14**.
84. Bahram, M., et al., *Vertical stratification of microbial communities in woody plants.* Phytobiomes Journal, 2022. **6**(2): p. 161-168.
85. Bálint, M., et al., *Host genotype shapes the foliar fungal microbiome of balsam poplar (Populus balsamifera).* PLoS ONE, 2013. **8**(1).
86. Põlme, S., et al., *Host preference and network properties in biotrophic plant–fungal associations.* New Phytologist, 2018. **217**(3): p. 1230-1239.
87. Kännaste, A., et al., *Impacts of Dutch elm disease–causing fungi on foliage photosynthetic characteristics and volatiles in Ulmus species with different pathogen resistance.* Tree Physiology, 2022. **00**: p. 1-18.
88. Agostinelli, M., et al., *Mycobiome of Fraxinus excelsior with different phenotypic susceptibility to ash dieback.* Frontiers in Forests and Global Change, 2021. **4**: p. 9-9.
89. González-Teuber, M., et al., *Leaf resistance traits influence endophytic fungi colonization and community composition in a South American temperate rainforest.* Journal of Ecology, 2020. **108**(3): p. 1019-1029.
90. Lee, D.-H., et al., *First report of Dutch elm disease caused by Ophiostoma novo-ulmi in South Korea.* Forests 2022, Vol. 13, Page 968, 2022. **13**(7): p. 968-968.
91. Klavina, D., et al., *Effect of stand thinning, former land use and individual tree parameters on wood inhabiting fungal community composition in young living Norway spruce.* Fungal Ecology, 2023. **65**: p. 101281.
92. Tedersoo, L., et al., *Regional-scale in-depth analysis of soil fungal diversity reveals strong pH and plant species effects in Northern Europe.* Frontiers in Microbiology, 2020. **Volume 11 - 2020**.

93. Abrego, N., et al., *Fungal communities decline with urbanization—more in air than in soil.* The ISME Journal, 2020. **14**(11): p. 2806-2815.
94. Butnor, J.R., et al., *Cold tolerance assay reveals evidence of climate adaptation among American elm (Ulmus americana L.) genotypes.* Forests, 2024. **15**(11).
95. Franić, I., et al., *Climate, host and geography shape insect and fungal communities of trees.* Scientific Reports 2023 13:1, 2023. **13**(1): p. 1-13.
96. Rusterholz, H.P. and B. Baur, *Changes in soil fungal diversity and composition along a rural–urban gradient.* Forests, 2023. **14**(11).
97. Christel, A., et al., *Urban land uses shape soil microbial abundance and diversity.* Science of the Total Environment, 2023. **883**.
98. Ranta, H. and I. Saloniemi, *Distribution of fungal foliage and shoot pathogens in a natural Scots pine population in relation to environmental variables.* Canadian Journal of Forest Research, 2005. **35**(3): p. 503-510.
99. Ghelardini, L. and A. Santini, *Avoidance by early flushing: a new perspective on Dutch elm disease research.* IForest, 2009. **2**(JULY): p. 143-153.
100. Krupinsky, J.M. and R.A. Cunningham, *Response of Siberian elm to inoculations with Spaeropsis ulmicola.* Plant Disease, 1993. **77**(7): p. 678-681.
101. Brodde, L., et al., *Diplodia sapinea as a contributing factor in the crown dieback of Scots pine (Pinus sylvestris) after a severe drought.* Forest Ecology and Management, 2023. **549**.
102. Blumenstein, K., et al., *Sphaeropsis sapinea and associated endophytes in Scots pine: interactions and effect on the host under variable water content.* Frontiers in Forests and Global Change, 2021. **4**: p. 55-55.
103. Gomzhina, M.M. and P.B. Gannibal, *Modern systematics of the genus Phoma sensu lato.* Mikologiya I Fitopatologiya, 2017. **51**(5): p. 268-275.
